# Reconciling observed and modeled estimates of urban terpenoid emissions

**DOI:** 10.1038/s41467-026-75075-9

**Published:** 2026-07-15

**Authors:** Yingnan Zhang, Kai Wu, Hui Wang, Eva Y. Pfannerstill, Sanjeevi Nagalingam, Matthew M. Coggon, Chelsea Stockwell, Viridiana M. Ruiz, Christina Ho, Ye Yu, Jared Novelly, Camille Pawlak, Saewung Kim, Carsten Warneke, Allen H. Goldstein, Alex B. Guenther

**Affiliations:** 1 Department of Earth System Science, University of California at Irvine, Irvine, CA USA; 2 Department of Civil and Environmental Engineering, University of California at Irvine, Irvine, CA USA; 3 Department of Environmental Science, Policy and Management, University of California at Berkeley, Berkeley, CA USA; 4 Chemical Sciences Laboratory, National Oceanic and Atmospheric Administration, Boulder, CO USA; 5 Department of Geography, University of California at Los Angeles, Los Angeles, CA USA; 6 Department of Civil and Environmental Engineering, University of California at Berkeley, Berkeley, CA USA; 7 Present Address: Nanjing-Helsinki Institute in Atmospheric and Earth System Sciences, Nanjing University, Suzhou, China; 8 Present Address: Institute of Climate and Energy Systems (ICE-3): Troposphere, Forschungszentrum Jülich, Jülich, Germany

**Keywords:** Atmospheric chemistry, Environmental impact

## Abstract

Extensive observations highlight the significance of terpenoids in urban air quality. However, emission frameworks fail to capture their magnitude and spatial variability, limiting air quality management and atmospheric modeling. Here we reconcile observed and modeled urban terpenoid emissions and uncover intense, underrepresented anthropogenic sources in Greater Los Angeles by combining improved emission inventories, airborne flux measurements, and ground-based mobile measurements. We demonstrate that an enhanced biogenic inventory significantly reduces the discrepancy between modeled and observed isoprene fluxes, and the remaining gap in monoterpenoids is explained predominantly by anthropogenic sectors. We identify that these emissions originate from previously overlooked food industry and commercial businesses, which exhibit a biogenic-like temperature dependence. These anthropogenic sources account for 44% of the top 10% observed monoterpenoid flux hotspots and contribute 17% of the total flux. Given their ubiquity in urban and industrialized regions worldwide, these poorly characterized sources may influence air quality beyond Los Angeles.

## Abstract (web summary)

Overlooked food-related and small commercial activities substantially contribute to terpenoid emissions in Los Angeles, helping explain gaps in current inventories and improving understanding of urban air pollution.

## Introduction

Air pollution remains a major environmental concern across urban and industrialized regions^1–3^, including many parts of the U.S.^4,5^. Since the Clean Air Act of 1970 and subsequent policy enhancements, the U.S. government has implemented stringent regulations to curb anthropogenic emissions of nitrogen oxides (NO_*x*_), volatile organic compounds (VOCs), and particulate matter (PM_10_ and PM_2.5_). While these efforts have led to substantial reductions in emissions, air pollution continues to pose risks to public health and the environment. Many U.S. cities, including Los Angeles (LA), still experience unhealthy levels of PM_2.5_ and ozone (O_3_)^5,6^.

A central challenge for cities like LA, where air pollution persists despite regulatory progress, is to identify the dominant sources of VOC emissions. During the early stages of rapid urban development, the industry and transportation sectors were the primary contributors to VOC emissions. As regulatory controls have reduced emissions from these sectors, the relative contribution of other sources has increased^7–12^. McDonald et al. (2018) highlights the increasing relative importance of volatile chemical products (VCPs), which now account for nearly half of fossil fuel-related VOC emissions in industrialized cities^9^. While these sources are primarily driven by population patterns, recent observations suggest that an additional class of urban VOC emissions is not fully captured by traditional source categories. In particular, temperature-sensitive VOC emissions, a feature usually considered biogenic, have been increasingly observed in urban environments^13,14^. For example, direct airborne flux measurements over LA demonstrate that terpenoid emissions, primarily isoprene and monoterpenoids (including monoterpenes and their oxygenated derivatives), show a strong temperature dependence and account for approximately 60% of total emitted VOC OH reactivity and secondary organic aerosol (SOA) formation potential during summer^14^.

Observational evidence across multiple cities further indicates that terpenoids are not exclusively biogenic but can also originate from anthropogenic activities such as traffic, VCPs, solvent use, and industrial processes^15–17^. This combination of anthropogenic origin and biogenic-like temperature dependence makes terpenoids challenging to attribute using conventional emission frameworks, in which anthropogenic emissions are typically treated as temperature-independent while biogenic emissions are parameterized as strongly temperature-dependent. As a result, these emissions are difficult to represent accurately in current emission inventories. For example, current major inventories, such as the California Air Resources Board (CARB) inventory, fail to capture the spatial variability of terpenoid emissions and substantially underestimate monoterpenoid emissions compared to airborne flux measurements^14^. Therefore, resolving this discrepancy requires not only identifying missing sources but also overcoming the limitations of current frameworks, which typically consider temperature-dependent emissions with biogenic origins. Addressing this mechanistic ambiguity is critical for effective air pollution control in LA and other similar regions.

In this study, we revisit urban terpenoid emissions by combining airborne flux observations, ground-based mobile measurements, and newly developed high-resolution emission inventories. We developed an enhanced biogenic emission inventory in LA using the Model of Emissions of Gases and Aerosols from Nature (MEGAN) version 3.2 with high-resolution vegetation datasets that integrate field measurements of urban trees and 10-m landcover maps. The enhanced biogenic inventory was combined with two widely used anthropogenic inventories, CARB_Anthro_ and GRA^2^PESv1.1^9,18–22^ (Greenhouse Gas and Air Pollutant Emissions System), which includes VCP, cooking, and other traditional known sources (Methods), and evaluated against airborne flux measurements at the grid-cell level. Our inventory explains the spatial variability and magnitudes of isoprene well, demonstrating the improved accuracy of the biogenic emission model, but underestimates the magnitude of monoterpenoid emissions. Moreover, using mobile laboratory measurements, we identified strong anthropogenic sources of monoterpenoids associated with the food industry and small commercial businesses that are not included in or are poorly represented in current inventories. These findings highlight that such sources contribute to air quality issues in LA and likely other dense urban centers beyond the current understanding of established source categories.

## Results

### Grid-cell comparison of terpenoid emissions between inventory and aircraft observations

**Fig. 1:**
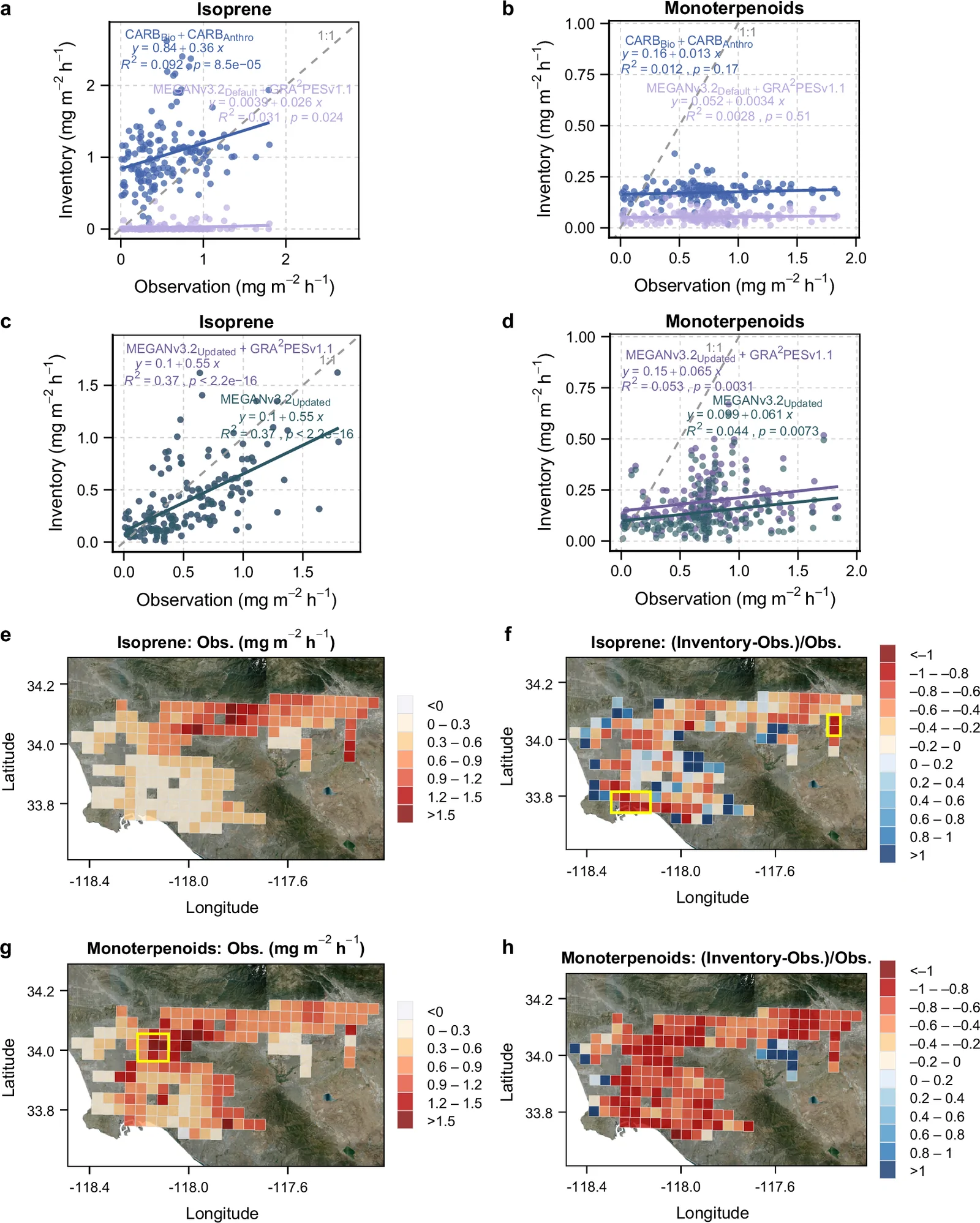
Evaluation of terpenoid emissions against airborne flux observations. **a**–**d** Scatterplots comparing inventory estimates with aircraft flux observations for (**a**, **c**) isoprene and (**b**, **d**) monoterpenoids. For (**a**, **b**), light purple points and lines indicate MEGANv3.2_Default_ + GRA^2^PESv1.1 inventories, while blue points and lines indicate CARB_Bio_ + CARB_Anthro_ inventories, reproduced following Pfannerstill et al. (2024)^14^. For (**c**, **d**), purple points and lines indicate MEGANv3.2_Updated_ + GRA^2^PESv1.1 inventories, while green points and lines represent the MEGANv3.2_Updated_ biogenic inventory. In **c**, the two regressions largely overlap, reflecting the minor contribution of anthropogenic emissions to isoprene and the dominance of biogenic sources. **e**, **g** Spatial distribution of aircraft-derived isoprene and monoterpenoid flux. Yellow squares highlight the Vernon and Commerce areas. **f**, **h** Relative bias between inventory and airborne observations, calculated as (Inventory − Observation)/Observation. Yellow squares highlight the Colton and Long Beach areas. For (**e**–**h**), background imagery is from the USDA National Agriculture Imagery Program.

Isoprene and total monoterpenoid emissions from inventories were evaluated against airborne flux measurements on a grid-to-grid (4 × 4 km^2^) and time-resolved basis. For each cell, emissions were calculated as the sum of anthropogenic emissions and MEGAN-estimated biogenic emissions (Methods) (Fig. 1a–d). We first evaluated two previously developed inventory frameworks (Fig. 1a, b), including the combined MEGANv3.2_Default_ + GRA^2^PESv1.1 and CARB_Bio_ + CARB_Anthro_ inventories. Under the MEGANv3.2_Default_ + GRA^2^PESv1.1 framework, both isoprene and monoterpenoid emissions were largely underestimated, explaining only 3% and 7% of observed values (0.54 ± 0.37 mg m^–2^ h^–1^ for isoprene; 0.73 ± 0.39 mg m^–2^ h^–1^ for monoterpenoids), respectively. In comparison, the CARB emission framework showed an overestimation of isoprene by 90% and an underestimation of monoterpenoids by 76%.

**Fig. 2:**
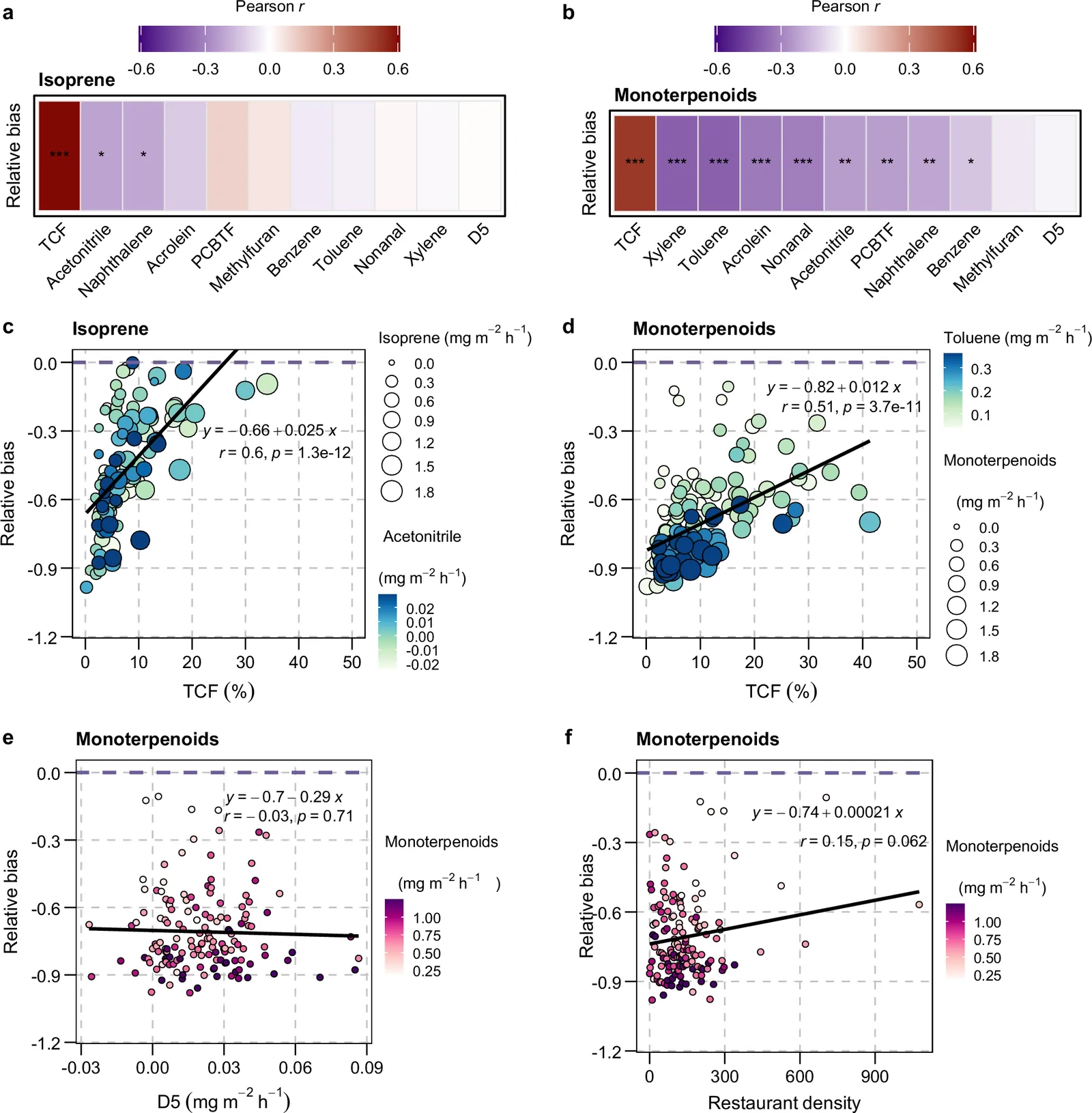
Relationship between relative bias of the combined MEGANv3.2_Updated_ + GRA^2^PESv1.1 terpenoid inventories and various emission indicators. **a**, **b** Pearson correlations between relative bias in (**a**) isoprene and (**b**) monoterpenoid fluxes and tree cover fraction (TCF, %) and airborne fluxes of selected chemical species. Colors indicate pearson correlation coefficients (*r*), and asterisks denote statistical significance (*p < 0.1; **p < 0.01; ***p < 0.001). **c**, **d** Isoprene relative bias and monoterpenoid relative bias versus TCF, colored by (**c**) acetonitrile flux and (**d**) toluene flux. Marker size denotes aircraft-derived isoprene and monoterpenoid flux, and marker color indicates the co-located emission proxy in each panel. **e**, **f** Monoterpenoid relative bias versus anthropogenic indicators, including (**e**) D5 flux and (**f**) restaurant density. Restaurant locations in Los Angeles were obtained via the Google Maps API, and grid-cell counts were used as a proxy for cooking intensity. Solid black lines represent least-squares fits, with *r* and *p*-values indicated.

We then evaluated the updated inventory developed in this study, which combines MEGANv3.2_Updated_ with GRA^2^PESv1.1 (Fig. 1c, d). Within this framework, biogenic sources accounted for nearly all isoprene emissions. Consistent with this, airborne flux measurements showed that high isoprene emissions were primarily associated with vegetated and remote areas (Fig. 1e), supporting the dominance of biogenic sources at the regional scale. The updated inventory reasonably captured the spatial distribution of isoprene (*R*^2^ = 0.37) and explained 74% (0.40 ± 0.33 mg m^–2^ h^–1^) of observed values, showing a substantial improvement compared with previous inventories (Fig. 1a). Similar results were derived when GRA^2^PESv1.1 was replaced with another commonly used anthropogenic inventory, CARB_Anthro_ (Supplementary Fig. 1), showing that the improvement arises mainly from refined biogenic inputs of urban trees and landcover maps. The remaining 26% inventory–measurement difference is relatively modest and within the uncertainty range of airborne flux measurements^14^. Nevertheless, we further examined whether the residual differences exhibited systematic source-related patterns (Figs. 1f, 2a, c, Supplementary Fig. 2). Larger negative biases were observed in several urban industrial areas, including Long Beach and Colton (yellow squares in Fig. 1f). In addition, isoprene relative bias showed significant negative correlations with acetonitrile (*r* = –0.22, *p* < 0.05) and naphthalene (*r* = –0.21, *p* < 0.05), tracers associated with combustion-related and mixed anthropogenic influences (Fig. 2a). These results suggest that unresolved urban anthropogenic influences may contribute locally to the residual discrepancy. This interpretation is consistent with previous gas chromatography (GC)-based observations in Houston showing anthropogenic isoprene enhancements in industrialized urban environments^23,24^.

In contrast, inventory estimates of monoterpenoids performed poorly (*R*^2^ = 0.053) and explained only 27% (0.19 ± 0.11 mg m^–2^ h^–1^) of observed values under MEGANv3.2_Updated_ + GRA^2^PESv1.1 framework. Analysis of flux patterns and relative bias ((Inventory − Observation)/Observation) (Fig. 1g, h) showed that, despite hotspots in remote forest areas, high monoterpenoid emissions were also observed in commercial and industrial areas of LA, such as Vernon and Commerce (yellow square in Fig. 1g), which inventories failed to capture.

We analyzed the relationship between relative bias in monoterpenoids and various emission indicators to identify sources contributing to inventory underestimation (Fig. 2, Supplementary Fig. 3). Toluene flux from airborne observations and tree cover fraction (TCF) were used as proxies for anthropogenic and tree-related biogenic emissions, respectively (Fig. 2b, d). TCF showed a significant positive correlation with relative bias (*r* = 0.51, *p* < 0.001), indicating better inventory performance in regions dominated by tree-related biogenic sources. In contrast, grid cells with strong monoterpenoid emissions that were largely underestimated by the inventory (relative bias < −60%) were typically associated with high toluene flux (≥ 0.3 mg m^–2^ h^–1^) and low TCF ( ≤ 15%) (Fig. 2d). The low TCF indicates that these emissions are unlikely to be dominated by tree-related biogenic sources. Moreover, none of the vegetation cover fractions, including tree, shrub, herbaceous, and crop types, showed significant negative correlations with relative bias (Supplementary Fig. 3), suggesting that biogenic sources were not major contributors to the inventory underestimation. The concurrent high toluene flux and their significant negative correlation with relative bias (*r* = –0.39, *p* < 0.001) points to unrepresented or poorly represented anthropogenic sources as the main contributors to both the high monoterpenoid emissions and their underestimation in the inventory.

Although previous studies indicate that proton-transfer-reaction time-of-flight mass spectrometry (PTR-ToF-MS) measurements of toluene may be subject to potential interferences^25^, we do not expect such contributions to alter our main interpretation that the missing monoterpenoid emissions are primarily anthropogenic, because this interpretation is supported by consistent relationships across multiple tracers and independent emission indicators. We further analyzed proxies for different anthropogenic activities. Decamethylcyclopentasiloxane (D5) flux and restaurant density were used as indicators of residential VCPs and restaurant cooking, respectively, two established anthropogenic sources of monoterpenoids (Fig. 2e, f)^26,27^. Neither showed a significant negative correlation with relative bias, suggesting that missing monoterpenoid emissions originate from anthropogenic activities beyond residential VCP and restaurant cooking sources. We also examined correlations between monoterpenoid relative bias and fluxes of other chemical species (Fig. 2b). Significant negative correlations were found with aromatic and oxygenated VOCs such as xylene (*r* = –0.39, *p* < 0.001), acrolein (*r* = –0.32, *p* < 0.001), and nonanal (*r* = –0.31, *p* < 0.001), suggesting that the missing monoterpenoid emissions are more strongly associated with solvent use (xylene)^17,27^ and lipid oxidation or heat-related processes (nonanal and acrolein)^28^.

### Anthropogenic monoterpenoid hotspots revealed by mobile measurements

Aircraft fluxes shown in the previous section capture emissions integrated over larger spatial scales. To complement these observations, we analyzed VOC concentrations from mobile-laboratory measurements in the LA basin to provide high-resolution information about near-surface sources and to identify missing sources of monoterpenoids. We focused on mobile measurements that fell within grid cells characterized by high toluene flux (≥ 0.3 mg m^–2^ h^–1^) from airborne measurements and low TCF ( ≤ 15%), to isolate areas strongly influenced by anthropogenic activities with minimal impact from biogenic emissions (Supplementary Fig. 4). These cells were highly heterogeneous, spanning commercial, residential, and industrial areas that included clusters of commercial business, industrial complexes, and major transportation corridors. Observed monoterpenoid flux within these cells ranged from 0.65 to 1.8 mg m^–2^ h^–1^ and accounted for 44% of top 10% flux measurements across LA, while relative bias indicated substantial underestimation (−92% to −76%) in current inventories due to missing sources.

**Fig. 3:**
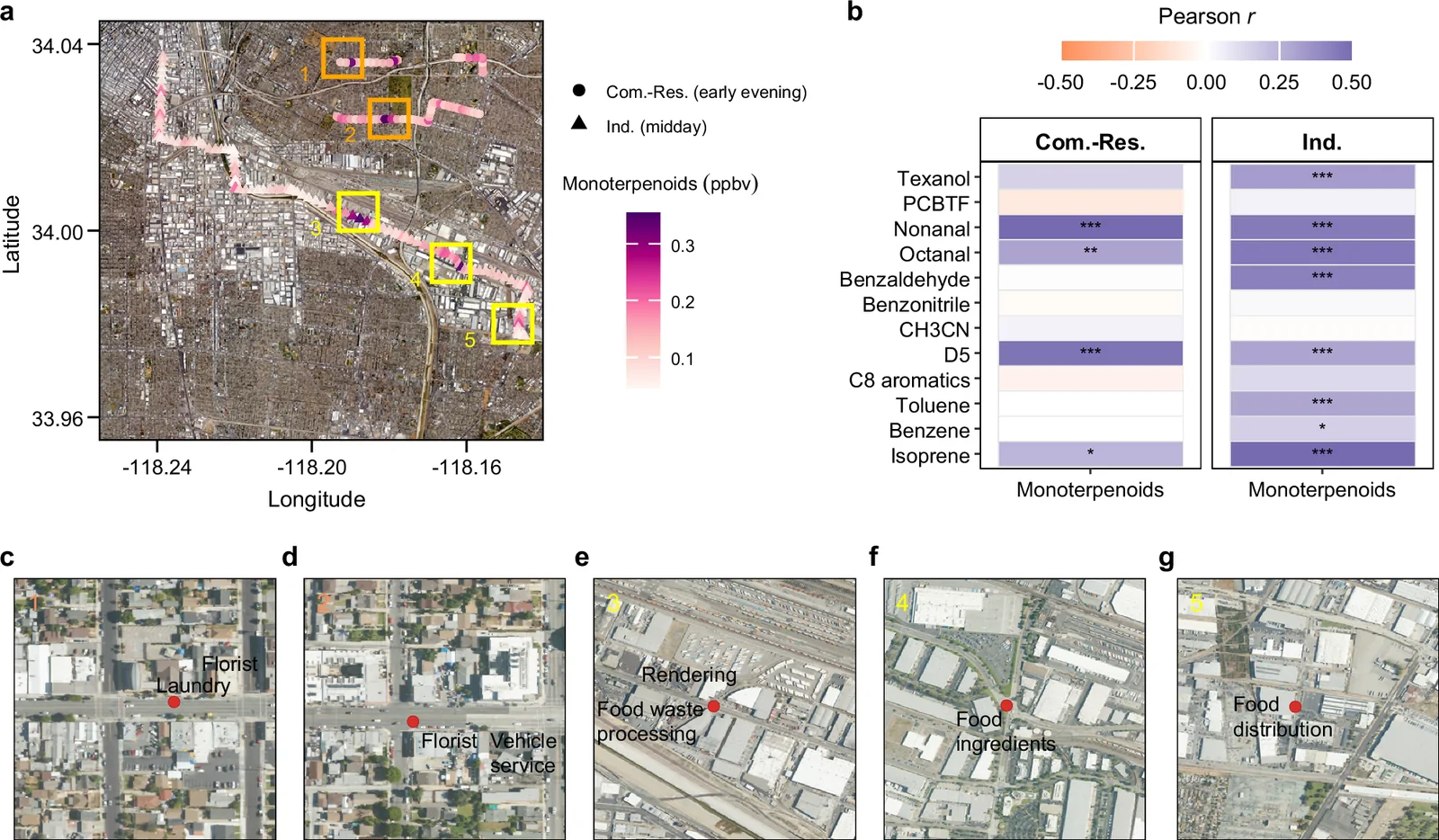
Spatial distribution of monoterpenoid concentrations and their correlations with major chemical species from mobile measurements. **a** Map showing the route colored by monoterpenoid concentrations; orange and yellow boxes mark five hotspots (≥ 0.2 ppbv). Marker shape denotes land-use type (Commercial-Residential or Industrial areas), with industrial areas characterized by white roofs. **b** Pearson correlations (*r*) between monoterpenoids and selected chemical species; asterisks denote significance (*p < 0.1; **p < 0.01; ***p < 0.001). **c–g** Aerial views of the five hotspot boxes in (**a**). Background imagery in (**a**) and (**c **− **g**) is from the USDA National Agriculture Imagery Program.

We examined the detailed geographic locations of mobile-identified monoterpenoid concentration hotspots (≥ 0.2 ppbv) to further investigate the missing sources (Fig. 3a, c–g). The mobile transects consisted of two routes, one conducted at midday (11:00–14:00 LT) across a light-industrial area (characterized by white-roofed facilities), and another around sunset (18:00 LT) across residential and commercial neighborhoods. In industrial clusters, monoterpenoid hotspots were observed near food processing (Fig. 3e), supply, and storage facilities (Fig. 3f, g), revealing food industry as a strong but unaccounted anthropogenic source. These industrial food processing facilities differ fundamentally from restaurant cooking activities, as they involve large-scale rendering, oil recycling, and food handling processes rather than on-site thermal cooking. Isoprene, octanal, and nonanal hotspots were also observed near these facilities (Supplementary Fig. 5), suggesting additional anthropogenic contributions from food industry activities. In commercial-residential clusters, monoterpenoid hotspots were frequently observed near laundry (Fig. 3c) and car wash facilities (Fig. 3d), which represent small commercial sources poorly captured by population-based VCP proxies. Additional hotspots near flower businesses highlight contributions from plant commercial activities (Fig. 3c, d) that are not included in current inventories. Ground-based mobile measurements suggest that inventory-unexplained monoterpenoid emissions in LA partially arise from a mix of unaccounted food industry sources, plant commercial activities, and the insufficiently resolved allocation of commercial VCP sources such as car decoration/wash business.

We compared mobile measurements in industrial and commercial-residential areas to assess how these anthropogenic sources manifest at the broader spatial scale (Fig. 3b). In industrial areas, monoterpenoid concentrations were strongly correlated with isoprene (*r* = 0.56, *p* < 0.001), octanal (*r* = 0.45, *p* < 0.001), nonanal (*r* = 0.44, *p* < 0.001), benzaldehyde (*r* = 0.43, *p* < 0.001), and texanol (*r* = 0.33, *p* < 0.001), suggesting co-emission with compounds associated with food processing (octanal, nonanal, and isoprene) (Supplementary Fig. 5) and industrial solvent (texanol)^26^. In contrast, in commercial-residential areas, monoterpenoid concentrations correlated strongly with D5 (*r* = 0.47, *p* < 0.001), a typical tracer of residential VCPs^26^, and with nonanal (*r* = 0.55, *p* < 0.001) and octanal (*r* = 0.30, *p* < 0.01), tracers for cooking activities^28^. These results indicate that residential VCP use and cooking dominated anthropogenic monoterpenoid emissions in commercial-residential areas, while food industry and solvent use dominated in industrial areas.

We acknowledge that the above results are derived from high-resolution mobile-laboratory measurements obtained in a limited area (Vernon and Commerce). Similar VOC measurements in other parts of Los Angeles would be needed to draw more robust conclusions regarding contribution of different anthropogenic sources to monoterpenoids across the entire region. Nonetheless, the combination of airborne flux measurements, which provide regional-scale constraints, and high-resolution mobile-laboratory observations, which enable local source identification, offers valuable complementary insights into terpenoid emission sources.

### Reconciliation of observed and modeled estimates of terpenoid emissions

Isoprene and monoterpenoid emissions were influenced by different sources, so we applied source-specific indicators to categorize grid cells and reconcile observed and modeled fluxes. Our above analyses indicated that isoprene emissions were dominated by biogenic sources, with additional contributions from urban industrial anthropogenic activities, whereas monoterpenoid emissions arose from a broader mix of biogenic and multiple anthropogenic sources, including food industry and commercial activities. Therefore, different tracer-based criteria were used to classify grid cells for isoprene and monoterpenoids, respectively.

For isoprene, we categorized grid cells as Biogenic-dominated and Mixed-source (Supplementary Fig. 6). In grid cells dominated by biogenic sources (defined as TCF ≥ 5% and acetonitrile flux ≤ 0.01 mg m^−2^ h^−1^ in Fig. 2c; 52% of all grid cells), the inventory explained 88% of observed isoprene fluxes (*R*^2^ = 0.44). In contrast, in mixed-source grid cells (remaining 48%), the explained fraction decreased to 55% (*R*^2^ = 0.23). This comparison indicates that residual discrepancies were more pronounced in mixed-source environments, where local anthropogenic influences may be less well represented by current inventories.

**Fig. 4:**
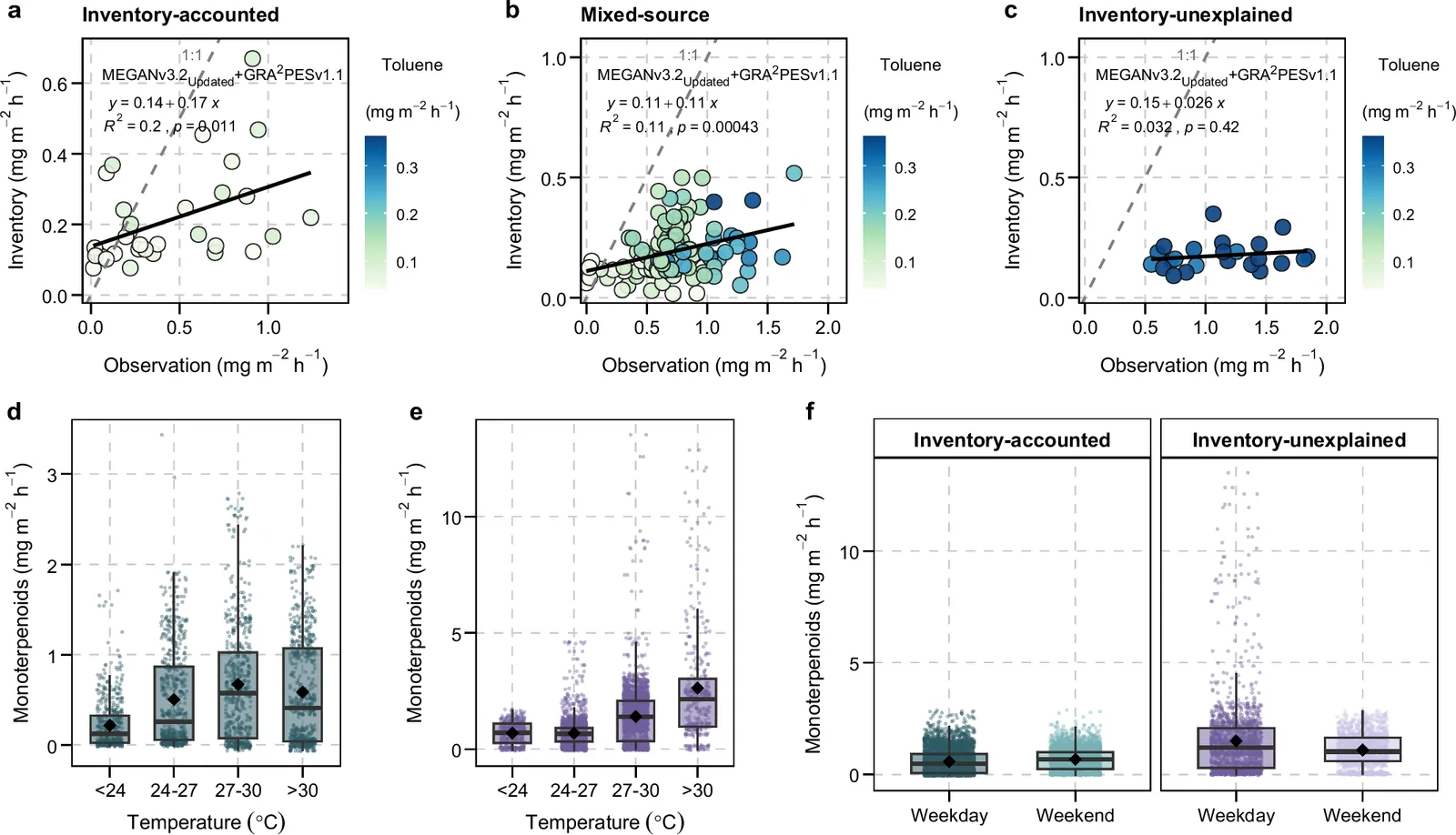
Comparison of monoterpenoid emissions in Inventory-accounted, Mixed-source, and Inventory-unexplained grid cells. **a**–**c** Scatterplots of inventory estimates (MEGANv3.2_Updated_ + GRA^2^PESv1.1) against airborne flux observations for (**a**) Inventory-accounted (toluene flux ≤ 0.1 mg m^–2^ h^–1^, tree cover fraction (TCF) ≥ 5%), (**b**) Mixed-source, and (**c**) Inventory-unexplained (toluene flux ≥ 0.3 mg m^–2^ h^–1^, TCF ≤ 15%) grid cells. **d, e** Temperature dependence of observed monoterpenoid flux in (**d**) Inventory-accounted and (**e**) Inventory-unexplained grid cells. **f** Weekday–weekend variations of observed monoterpenoid flux in Inventory-accounted and Inventory-unexplained grid cells. For (**d**–**f**), dots represent individual observations, boxes show the median line and the lower and upper quartiles, whiskers extend to 1.5×interquartile range (IQR), and diamonds denote means.

For monoterpenoids, we categorized grid cells as Inventory-accounted (biogenic-, VCP-, cooking-, and other known sources-dominated, which are accounted for within the combined MEGANv3.2_Updated_ and GRA^2^PESv1.1 inventories), Inventory-unexplained (strongly affected by missing or underrepresented anthropogenic sources), and Mixed-source (influenced by both inventory-accounted and unexplained sources) to refine inventory evaluations (Fig. 4, Supplementary Fig. 7). Inventory-accounted grid cells were defined as those with toluene flux ≤ 0.1 mg m^–2^ h^–1^ and TCF ≥ 5% (20% of all grid cells), while Inventory-unexplained grid cells were characterized by TCF ≤ 15% and toluene flux ≥ 0.3 mg m^–2^ h^–1^ (14% of all grid cells). The remaining 66% grid cells were classified as Mixed-source type. This categorization allowed us to assess the performance of current inventories across different source sectors and to gain insights into improving emission estimates.

Based on airborne flux measurements, the product of occupied grid-cell counts and mean flux in each category (normalized by the total fluxes across all grid cells) indicated that Inventory-accounted, Mixed-source, and Inventory-unexplained contributed 12%, 67%, and 20% of the total monoterpenoid flux across LA, respectively (Supplementary Fig. 7). In Inventory-accounted and Mixed-source grid cells, the inventory estimates explained 49% (*R*^2^ = 0.20) and 27% (*R*^2^ = 0.11) of observed monoterpenoid fluxes (Fig. 4a, b), which indicates that the current inventory could capture some spatial variability but still underestimated the magnitude. In contrast, in Inventory-unexplained grid cells, inventory estimates exhibited poor performance in both spatial distribution (*R*^2^ = 0.032) and magnitude (Fig. 4c). Only 16% of fluxes were captured by our inventories, while the remaining 84% likely originate from missing or underrepresented anthropogenic sources. This implies that previously unaccounted anthropogenic sources contributed ≥17% of the total monoterpenoid flux across LA (Supplementary Fig. 7). As demonstrated in previous sections, these unexplained emissions likely come from unaccounted food industry sources, plant commercial activities, and insufficiently resolved allocations of commercial VCP sources.

Although comprising only 14% of all grid cells, the Inventory-unexplained sources exhibited higher monoterpenoid emission intensities than those dominated by known sources. The observed peak flux in Inventory-unexplained grid cells (14 ± 1.6 mg m^–2^ h^–1^; ±uncertainty) was more than 4 times greater than that in Inventory-accounted grid cells (3.4 ± 1.6 mg m^–2^ h^–1^; ±uncertainty) (Fig. 4f). Similar to the Inventory-accounted category, the Inventory-unexplained category also exhibited a strong temperature dependence (Fig. 4d, e), effectively mimicking the emission behavior of vegetation. However, the anthropogenic nature of these sources is clearly confirmed by the marked weekday–weekend variations in emission intensity (Fig. 4f), a temporal signature absent in biogenic emissions. These results highlight the need to explicitly incorporate both the activity-driven temporal variability and temperature dependence of these sources into current emission inventories and air quality management frameworks.

### Implications and outlook

Recent studies have highlighted the important role of terpenoid emissions in urban air quality, particularly in the context of a warming climate^14,29,30^. Conventionally, these emissions are attributed to biogenic sources, VCP use, and cooking activities. By integrating grid-cell information from refined inventories with airborne flux and ground-based mobile measurements, we identified distinct additional anthropogenic sources from the food industry and small commercial activities, such as florists, laundry, and car wash facilities. These emissions appear locally intense and exhibit temperature sensitivity that may resemble biogenic behavior under certain conditions, potentially complicating source attribution if not properly represented in emission inventories.

Understanding the sources, activity profile, and temperature sensitivity of terpenoid emissions offers guidance for urban air quality management. While recent studies have highlighted the climate penalty of temperature-driven anthropogenic emissions^13^, our findings point to a broader limitation in current emission frameworks in that temperature-dependent processes in certain anthropogenic sectors may not be adequately captured. This limitation may also extend to other plausible sources, such as asphalt-related emissions from road pavement, which have been shown to increase with temperature and could contribute to urban air pollution^31,32^. Re-attributing these hidden anthropogenic emissions and incorporating both activity-related temporal variability and temperature dependence into emission inventories may improve the representation of anthropogenic VOC emissions. In particular, distinguishing between temperature-independent, temperature-dependent, and strongly temperature-dependent anthropogenic emissions could help improve the representation of urban VOC emissions in chemical transport models under a changing climate.

Moreover, these terpenoid hotspots are concentrated in densely populated urban areas co-located with heavy traffic. In these environments, terpenoids may react rapidly with hydroxyl radicals under high NO_*x*_ conditions, potentially enhancing O_3_ and SOA formation and contributing to local air pollution. Given that food industry and commercial activities are intrinsic to urban environments, better characterization of these sectors may provide additional opportunities to improve the representation of VOC emissions and their impact on urban air quality.

Our results also provide insights into refining the biogenic emission model. The substantial improvement in isoprene estimates after updating urban vegetation inputs demonstrates the importance of accurately representing urban land cover and vegetation composition. However, residual inventory–measurement differences remain, especially for monoterpenoids. Although these differences cannot be attributed solely to biogenic emissions, they highlight remaining uncertainties in urban biogenic VOC (BVOC) modeling, including canopy microenvironment, emission factors, and missing processes such as vegetation stress responses and non-foliar emissions. First, urban vegetation is exposed to a more complex thermal and light environment than rural areas, suggesting that the canopy microenvironment model may not accurately represent light and temperature variations within urban environments. Additionally, BVOC emission models typically assume herbaceous plants have low isoprene and monoterpene emissions. However, Wang et al. (2024) demonstrated that sedges, a common herbaceous plant in urban areas, can emit substantial isoprene under high temperatures^33^. Uncertainties in tree and shrub emission factors further contribute to this underestimation. Beyond measurement-related uncertainties, non-foliar plant structures (e.g., bark, seeds, and flowers) can also emit BVOCs^34–36^, yet their contributions are not represented in current models. Finally, urban vegetation experiences greater environmental stress, including heat and air pollution (e.g., ozone exposure), which can affect BVOC emissions^37,38^. For example, heat stress can trigger monoterpene bursts from Cupressaceae species in urban settings^37^. However, these stress-induced emissions are poorly represented in existing models. While many of these effects have been observed in laboratory studies, additional in-situ observations are needed to assess their real-world significance and accurately represent the controlling processes in BVOC emission models.

## Methods

### CARB biogenic inventory

The CARB 2021 biogenic inventory was generated using MEGANv3.0. For the CARB inventory, the default MEGAN3.0 datasets for plant growth form, ecotype, and emission factors are used. Leaf area index (LAI) for non-urban grid cells is based on the year-specific 8-day 500-m resolution MODIS Terra/Aqua combined product (MCD15A2H). Because the MODIS LAI product does not provide information for urban regions, urban LAI is estimated from the US Forest Service’s Forest Inventory and Analysis (FIA) urban tree plot data, processed using i-Tree v6. Peak summertime urban LAI for different urban regions is estimated, and this peak value is then adjusted for each 8-day MODIS period based on the relative change in non-urban MODIS LAI across the state. Hourly meteorology inputs used to drive MEGANv3.0 simulations are provided by Weather Research & Forecast (WRF) model simulations, and stress factor adjustments are not considered.

### Development of high-resolution biogenic emission inventory using MEGANv3.2

In this study, we use MEGANv3.2 for biogenic emission simulations. MEGANv3.2 follows the same modeling framework as earlier versions (e.g., MEGANv3.0). The general framework of MEGAN (applicable to v3.0 and later versions) can be expressed as:

1 $$F=\varepsilon \times {{{\rm{LAI}}}}\times \gamma$$

where *F*, *ε*, LAI and *γ* represent the flux amount, the standard grid-cell average emission factor, leaf area index and the total emission activity factor, respectively. The grid-cell average emission factors, *ε*, are derived as:

2 $${\varepsilon }_{{{{\rm{Grid}}}}}=\sum {f}_{{{{\rm{GF}}}}}\times \sum {f}_{v}\times {\varepsilon }_{v}$$

where *f*_GF_, *f*_*v*_, and *ε*_*v*_ denote the fraction of growth form (GF), the fraction of vegetation species or types within each Ecotype, and leaf-level emission factors of vegetation species or types, respectively. GF represents the spatial distribution of four major vegetation groups: Tree, Shrub, Herb, and Crop. The Ecotype map layer and associated Vegetation Composition Look-Up Table (VCLUT) are used to calculate the average emission factors for each GF group in each ecotype. In this framework, regions covered by the same VCLUT type are assigned an Ecotype code, and the emission factor for each GF group within the same Ecotype is derived from the fraction of vegetation species (*f*_*v*_) and corresponding leaf-level emission factors (*ε*_*v*_) as presented in Eq. (2). Finally, the gridded emission factor is calculated as the weighted averaged emission factors of the four GF groups in the same grid cell.

The activity factor, *γ*, accounts for the impact of temperature, light, leaf age, water stress, ozone stress, heat stress, cold stress, wind stress, and CO_2_ concentration on BVOCs. The weather inputs for calculating the activity factor came from the hourly output of WRF model version 4.1.1^39^, which is described in the following section. We did not consider the impact of water and other stresses on BVOCs.

We performed MEGANv3.2 simulations with default vegetation inputs (i.e., the standard MEGANv3.2 configuration, MEGANv3.2_Default_) and updated inputs (i.e., MEGANv3.2_Updated_) to quantify the effects of vegetation input updates (Fig. 1). The comparison between these two simulations indicates that the improvement in BVOC emission estimates arises mainly from refined biogenic inputs of urban trees and landcover maps.

### Updated vegetation inputs for MEGANv3.2 simulations

LAI inputs were derived from the Copernicus Global Land Service (CGLS) Collection 300-m datasets (https://land.copernicus.eu/en/products/vegetation/leaf-area-index-300m-v1.0). For urban landscapes in the study region, ecotypes and tree species composition were derived from a zip-code–level urban tree inventory for California^40^ (https://lookerstudio.google.com/reporting/880d448d-de26-48d3-b563-0c6317e456e4/page/jWHKB) and used to construct the MEGAN ecotype map layer and associated VCLUT. Growth form fraction inputs were derived from WorldCover 10-m data, where grid cells dominated by a specific vegetation type were assigned a fraction of 100%, while all others were set to 0%. To validate the accuracy of WorldCover data, we compared city-average vegetation cover fraction and tree cover fraction in major California cities against estimates from i-Tree (https://www.itreetools.org/) and Google Environmental Insights Explorer (GEIE; https://insights.sustainability.google/) virtual observations (Supplementary Fig. 8). The strong agreement with field measurements supports the use of WorldCover 10-m data as a GF input for MEGANv3.2.

### WRF meteorology for MEGANv3.2 simulations

The WRF model v4.1.1 was adopted to simulate meteorological fields across California in June 2021, with a total of 31 vertical layers extending from the surface to 100 hPa. Three nested domains are applied in WRF, with horizontal resolutions of 36 km, 12 km, and 4 km, respectively. The broadest outer domain encompasses the western U.S., and the innermost domain specifically focuses on California. The simulation of WRF is initialized on 27^th^ May 00:00 UTC and ended on 30^th^ June 23:00 UTC, 2021. The North American Regional Reanalysis (NARR) dataset was used to provide initial and boundary conditions for the WRF model^41^. For vegetation and land use inputs to the WRF model, the simulation incorporates the 2011 National Land Cover Database (NLCD), which provides 40-category land-cover classification for U.S. To minimize the impacts of initial conditions, simulation results prior to 1^st^ June, 00:00 UTC were discarded as spin-up. The physical parameterization schemes selected in WRF were consistent with Wu et al. (2023)^42^, which is based on best practices of sensitivity experiments that yield the greatest model performance^43^.

### CARB and GRA^2^PESv1.1 anthropogenic inventories

The CARB 2021 anthropogenic inventory includes emissions from point, area, and mobile sources. Mobile emissions were estimated using the EMFAC2021 and OFFROAD models, while stationary sources were derived from facility-reported surveys combined with emission factors from the California Air Toxics Emission Factor (CATEF) database.

The GRA^2^PESv1.1 inventory integrates datasets from the U.S. Energy Information Administration (EIA) and Environmental Protection Agency (EPA), together with several sector-specific inventories, including the Fuel-based Oil and Gas (FOG), Fuel-based Inventory of Vehicle Emissions (FIVE), and Volatile Chemical Products (VCP) inventories.

Both CARB and GRA^2^PESv1.1 anthropogenic inventories are available at 4-km spatial resolution across California, but their grid structures differ. The CARB inventory aligns with our MEGANv3.2 grids, while the GRA^2^PESv1.1 inventory was interpolated onto the MEGAN grids. We extracted isoprene and monoterpene emission data from these inventories and added them to the MEGAN_Updated_ biogenic inventory to form the total emission estimates evaluated against airborne flux measurements.

### Airborne flux measurement

Flux measurements were derived using airborne eddy covariance onboard a Twin Otter aircraft equipped with a Vocus PTR-ToF-MS (Aerodyne Inc., Billerica, MA, USA) for VOCs during June 2021^14^. During the flights, the PTR-ToF-MS was operated at a reactor temperature of 60 °C, pressure of 2.0 mbar, and E/N of ca. 130 Td. Key ions, likely compound assignments, and potential interferences for the PTR-ToF-MS measurements are provided in Supplementary Methods. Flight segments were carefully selected based on stability criteria, including consistent altitude and minimal variations in aircraft roll and pitch. VOC concentrations were aligned with high-frequency vertical wind measurements, and fluxes were calculated using a continuous wavelet transform, which decomposed the covariance signal into time and frequency domains. Corrections were applied for chemical vertical flux divergence due to oxidation losses, and for physical vertical divergence caused by advection and entrainment, using measured flux gradients. Data quality was ensured through calibration with gas standards, uncertainty quantification via Monte Carlo error propagation, and filtering of segments with excessive turbulence or short length.

For comparison with emission inventories, each footprint (corresponding to a measured flux) was spatially matched to overlapping inventory grid cells^27^. Footprints were retained if their overlap exceeded 10% of the grid cell area and if the cumulative overlaps covered at least 100% of the footprint. Observations were temporally matched to inventory estimates by hour and date, and weighted by spatial overlap. For consistency with MEGANv3.2 simulations, total monoterpenoid flux was calculated by summing the fluxes of ions identified as C_10_H_17_^+^, C_10_H_17_O^+^, C_10_H_19_O^+^, and C_10_H_21_O^+^, corresponding to monoterpenes and their oxygenated derivatives.

### Mobile-laboratory measurements

Mobile measurements of VOCs were conducted in LA (eight drive days in August–September 2021) using the National Oceanic and Atmospheric Administration (NOAA) Chemical Sciences Laboratory (CSL) mobile laboratory. The platform was equipped with a suite of instruments for characterizing reactive gases and particles, including a Vocus PTR-ToF-MS for real-time VOC quantification. The PTR-ToF-MS sampled air from the mobile laboratory through a 1 m Teflon tube and measured VOC mixing ratios at 1 Hz time resolution. Instrument backgrounds were determined every 15 min by passing air through a platinum catalyst heated to 350 °C. Each species was calibrated using gravimetrically prepared gas standards^44^.

In this study, we focused on mobile measurements within grid cells strongly affected by anthropogenic activities but minimally affected by biogenic emissions, defined by high toluene flux (≥ 0.3 mg m^–2^ h^–1^) from airborne observations and low TCF ( ≤ 15%). We analyzed mobile measurements from two targeted transects (Fig. 3a): one conducted during daytime (11:00–14:00 LT) on 4 August 2021, traversing a light industrial area, and another performed around sunset (18:00 LT) on 6 August 2021 across residential and commercial neighborhoods.

## Supplementary information

Supplementary Information

Transparent Peer Review file

## Data Availability

The data generated in this study to reproduce the figures and main analyses have been deposited in Zenodo under DOI 10.5281/zenodo.20548485^45^. The GRA^2^PESv1.1 anthropogenic emission inventories, airborne flux measurements and mobile-laboratory measurement data are available from the NOAA CSL SUNVEx project website at https://csl.noaa.gov/projects/sunvex/. The CARB terpenoid emission inventories used in this study were provided by the Regional Air Quality Modeling Section, Modeling and Meteorology Branch of the California Air Resources Board (CARB). These CARB data are available under restricted access because redistribution is controlled by the data provider. Access requests should be directed to CARB through its data request procedures. All other data supporting the findings in this study are included in the manuscript and/or SI Appendix.

## Code availability

The codes generated in this study to reproduce the figures and main analyses have been deposited in Zenodo under 10.5281/zenodo.20548485^45^.
