# Supplementary material for: Reconciling observed and modeled estimates of urban terpenoid emissions
Source: Nat Commun. 2026 Jul 15;17:8671. doi: 10.1038/s41467-026-75075-9 (PMC13490599; doi:10.1038/s41467-026-75075-9)
Supplement: Supplementary file 1 [file 41467_2026_75075_MOESM1_ESM.pdf]

Supplementary Materials for

Reconciling observed and modeled estimates of urban terpenoid emissions

Yingnan Zhang[1], Kai Wu[2], Hui Wang[1*,†], Eva Y. Pfannerstill[3†], Sanjeevi Nagalingam[1], Matthew M. Coggon[4], Chelsea Stockwell[4], Viridiana M. Ruiz[1], Christina Ho[1], Ye Yu[1], Jared Novelly[1], Camille Pawlak[5], Saewung Kim[1], Carsten Warneke[4], Allen H. Goldstein[3,6], Alex B. Guenther[1*]

[1] Department of Earth System Science, University of California, Irvine, CA, USA

[2] Department of Civil and Environmental Engineering, University of California, Irvine, CA, USA

[3] Department of Environmental Science, Policy and Management, University of California, Berkeley, CA, USA

[4] Chemical Sciences Laboratory, National Oceanic and Atmospheric Administration, Boulder, CO, USA

[5] Department of Geography, University of California, Los Angeles, CA, USA

[6] Department of Civil and Environmental Engineering, University of California, Berkeley, CA, USA

[*]Corresponding author. Email: huiw16@uci.edu (H.W.); alex.guenther@uci.edu (A.B.G.)

[†]Present address:

Nanjing-Helsinki Institute in Atmospheric and Earth System Sciences, Nanjing University, Suzhou, China (Hui Wang);

Institute of Climate and Energy Systems (ICE-3): Troposphere, Forschungszentrum Jülich, Jülich, Germany; Institute of Geophysics and Meteorology, University of Cologne, Cologne, Germany (Eva Y. Pfannerstill)

The file includes:

Supplementary Methods

Supplementary Figures 1–8

Supplementary References

Supplementary Methods

Key ions and potential sources of interference from Vocus PTR-ToF-MS measurements during airborne flux measurements. For m/z 69 ($C5H8H^+$), interference from anthropogenic VOCs such as cycloalkanes and long-chain aldehydes were accounted for explicitly[1]. The correction approach of Coggon et al. (2024)[2] was applied, in which m/z 69.0699 was adjusted based on its relationship with fragment ions m/z 111.117 and 125.132 derived from nonanal calibration and observations over oil and gas fields in the San Joaquin Valley[1]. While this correction affected mixing ratios in the urban background, its impact on fluxes was small (10%), indicating that most of the covariance signal reflects true isoprene emissions.

For the monoterpene ion (m/z 137.13), potential contributing species were also evaluated[1]. This ion is referred to here as a monoterpenoid signal because it can include contributions from both non-functionalized monoterpenes and fragments from oxygenated monoterpenoids or monoterpene alcohols with parent mass m/z 155.14 (e.g., eucalyptol, linalool, cineole, terpineol)[3]. However, interference from sesquiterpene fragmentation is expected to be negligible, as only 5% of sesquiterpenes fragment to m/z 137.13 and their concentrations and fluxes were about an order of magnitude lower than monoterpenes, implying an interference below 1%[1].

In addition, VOCs shown individually in the figures, e.g., isoprene, monoterpenes (mixture of α- and β-pinene and limonene), acetonitrile, acrolein, benzene, toluene, xylene, D5 siloxane, nonanal, and furan, contributing substantially to the fluxes, or driving inventory discrepancies were gas-standard calibrated and/or have well-characterized fragmentation behavior[2,4], such that remaining interferences are expected to be small.

Supplementary Figures

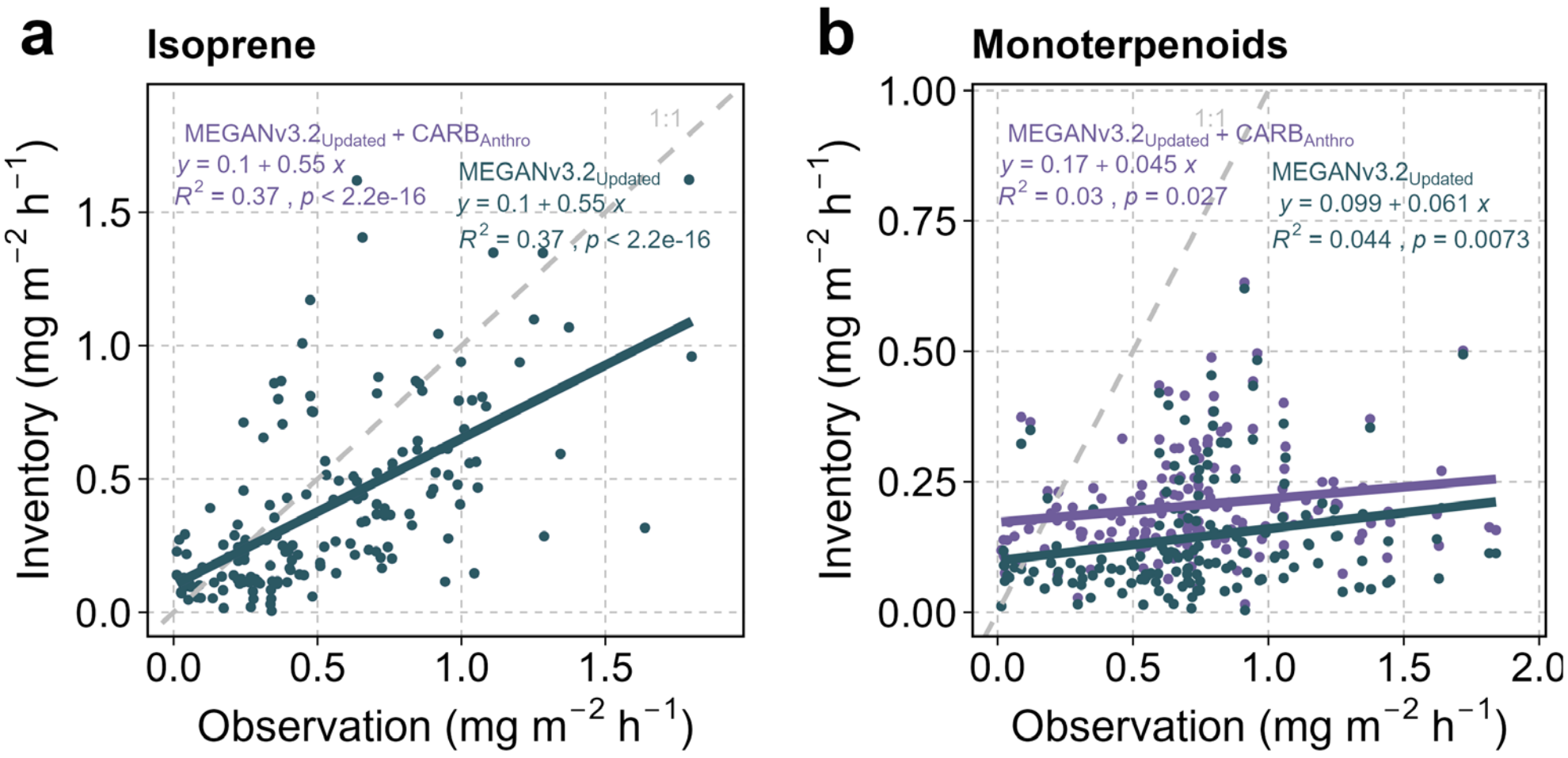

Supplementary Fig. 1. Evaluation of terpenoid emissions against airborne flux measurements. a Isoprene fluxes. **b** Monoterpenoid fluxes. Green points and regression lines represent the MEGANv3.2 biogenic inventory (MEGANv3.2$_{\text{Updated}}$); purple points and regression lines indicate the combined MEGANv3.2$_{\text{Updated}}$ + CARB$_{\text{Anthro}}$ inventories.

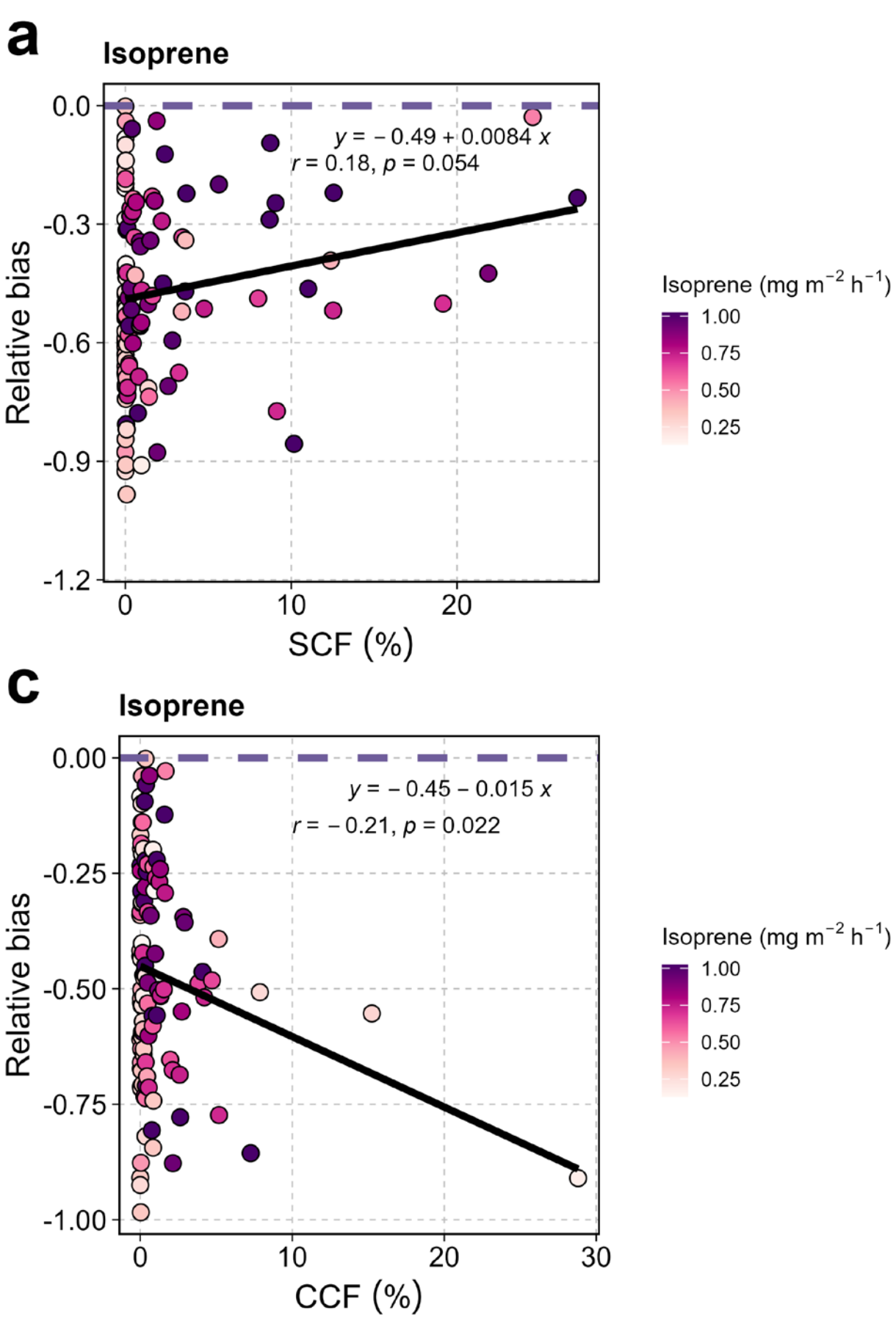

Supplementary Fig. 2. Relationship between relative bias in isoprene flux and non-tree biogenic sources. a Shrub cover fraction (SCF, %). **b** Herbaceous cover fraction (HCF, %). **c** Crop cover fraction (CCF, %). Marker color denotes aircraft-derived isoprene flux. Solid black lines indicate least-squares fits, with Pearson *r* and *p* values provided.

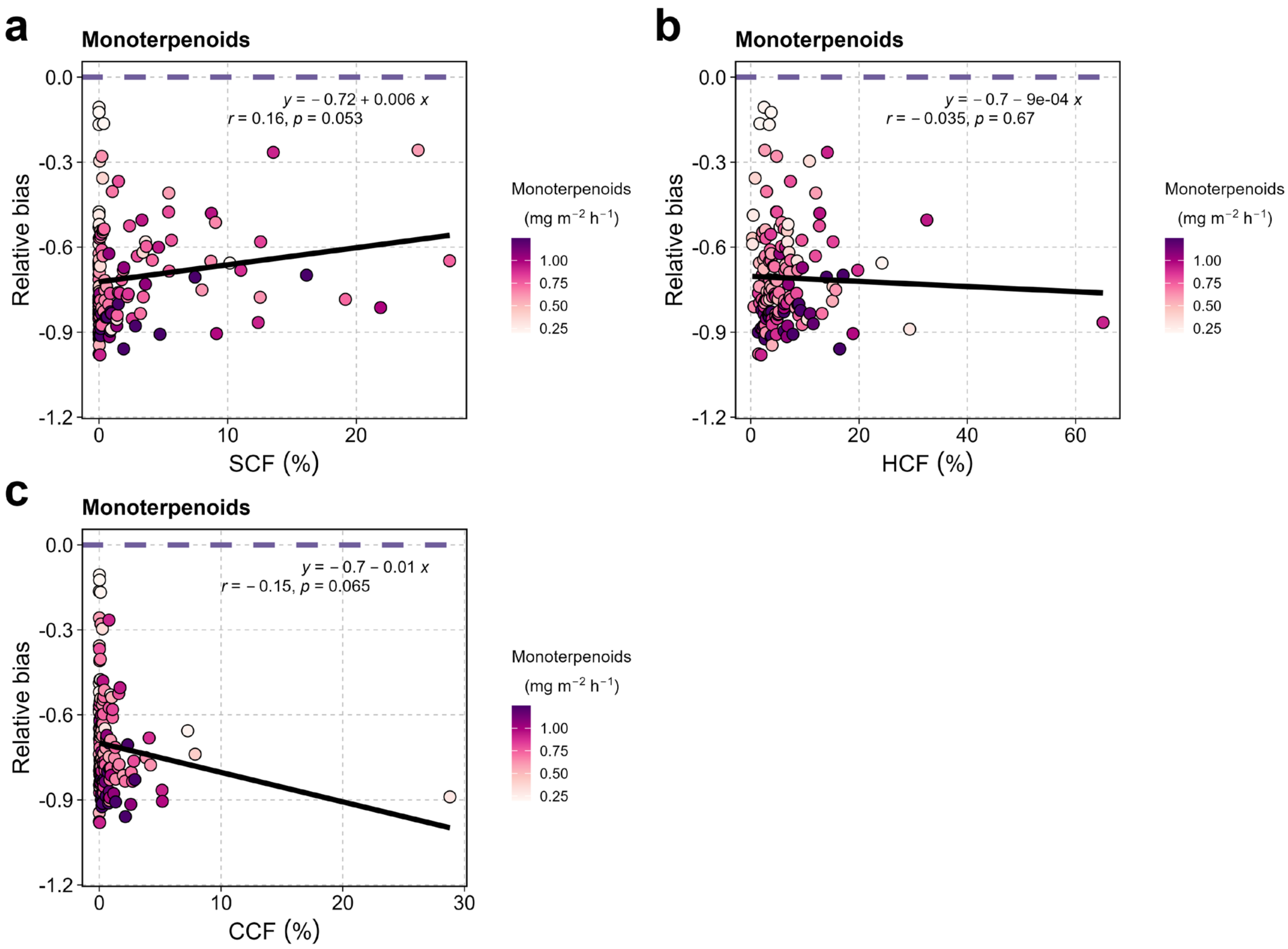

Supplementary Fig. 3. Relationship between relative bias in monoterpenoid flux and non-tree biogenic sources. a Shrub cover fraction (SCF, %). **b** Herbaceous cover fraction (HCF, %). **c** Crop cover fraction (CCF, %). Marker color denotes aircraft-derived monoterpenoid flux. Solid black lines indicate least-squares fits, with Pearson *r* and *p* values provided.

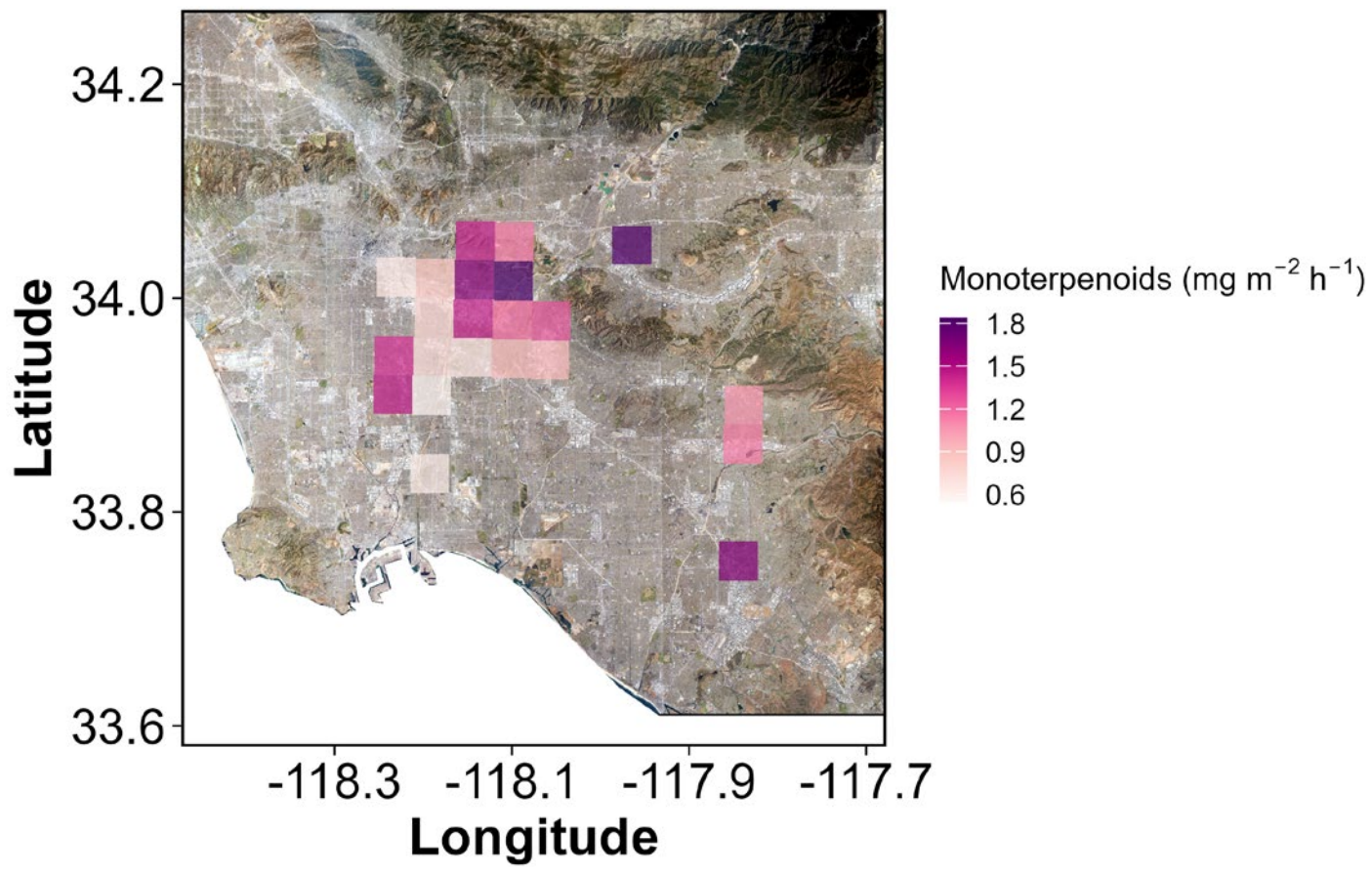

Supplementary Fig. 4. Spatial distribution of grid cells strongly affected by anthropogenic sources. Grid cells were identified using the following criteria: tree cover fraction (TCF) ≤ 15% and toluene flux ≥ 0.3 mg m^{-2} h^{-1}. Background imagery is from the USDA National Agriculture Imagery Program.

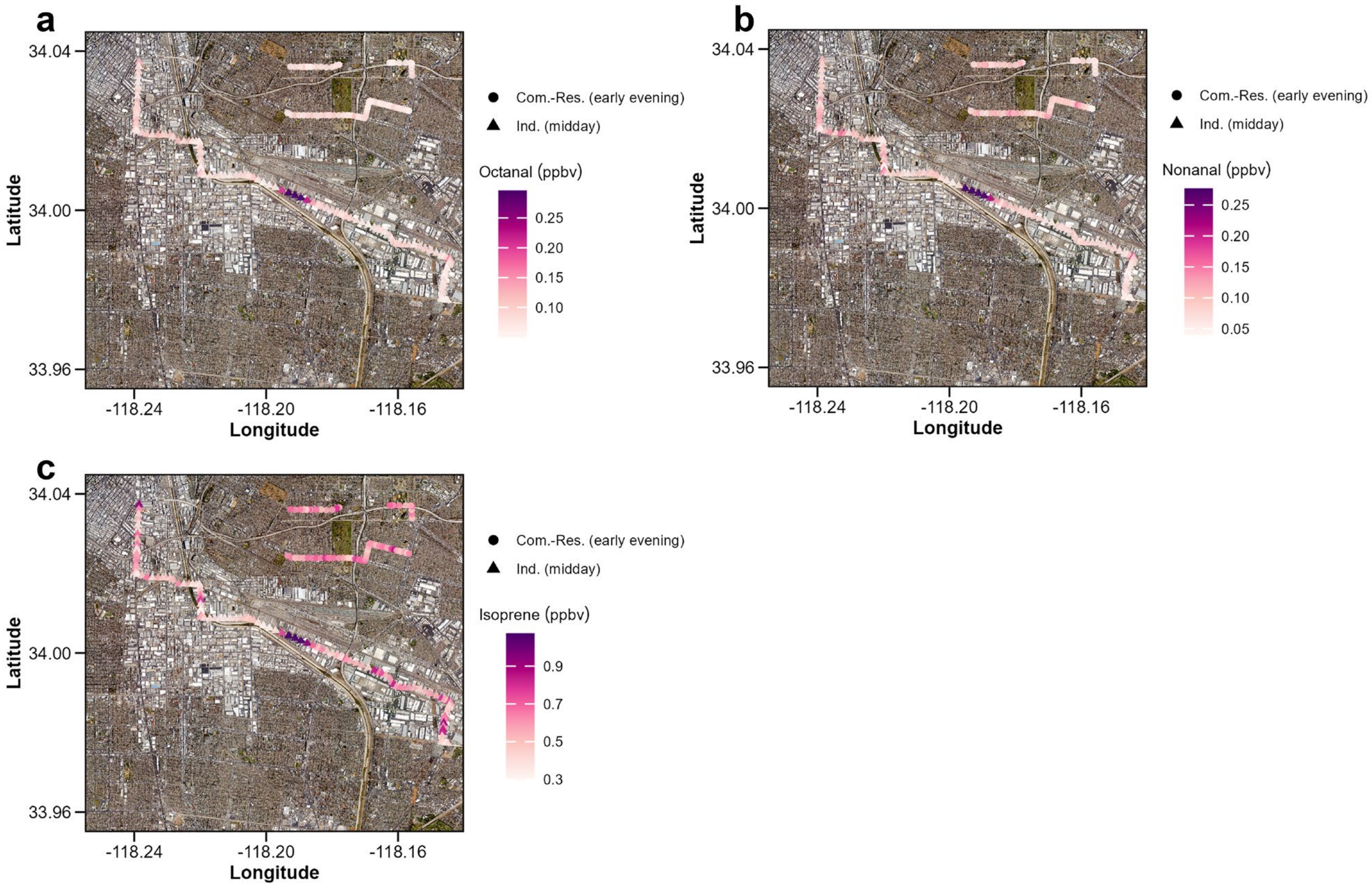

Supplementary Fig. 5. Spatial distribution of octanal, nonanal, and isoprene concentrations from mobile-laboratory measurements. a Octanal. **b** Nonanal. **c** Isoprene. Marker shape denotes land-use type, with industrial areas characterized by white roofs. Background imagery is from the USDA National Agriculture Imagery Program.

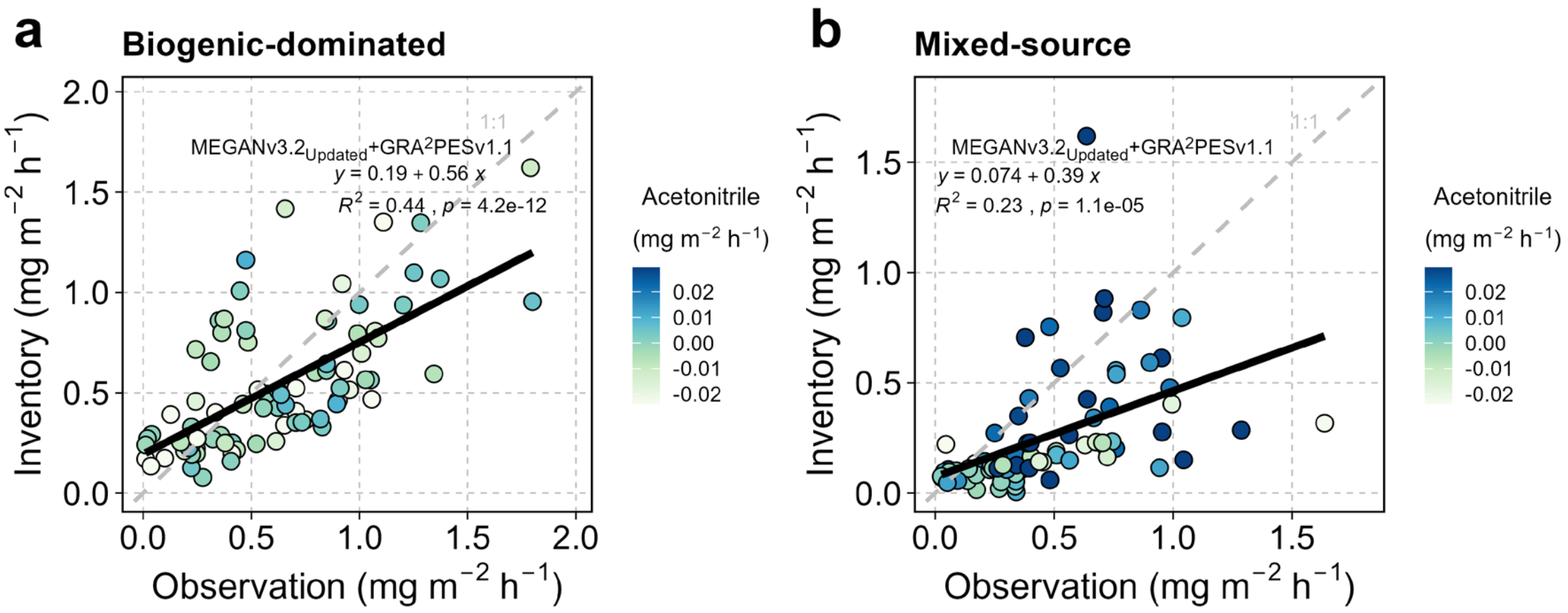

Supplementary Fig. 6. Evaluation of isoprene emissions against airborne flux measurements in Biogenic-dominated and Mixed-source grid cells. a Biogenic-dominated grid cells, defined by acetonitrile flux ≤ 0.01 mg m^{-2} h^{-1} and tree cover fraction (TCF) ≥ 5%. **b** Mixed-source grid cells. Inventory estimates are from MEGANv3.2$_{Updated}$ + GRA^{2}PESv1.1 and are compared with airborne flux observations. Points are colored by acetonitrile flux. The black solid line represents the linear regression, and the grey dashed line denotes the 1:1 relationship.

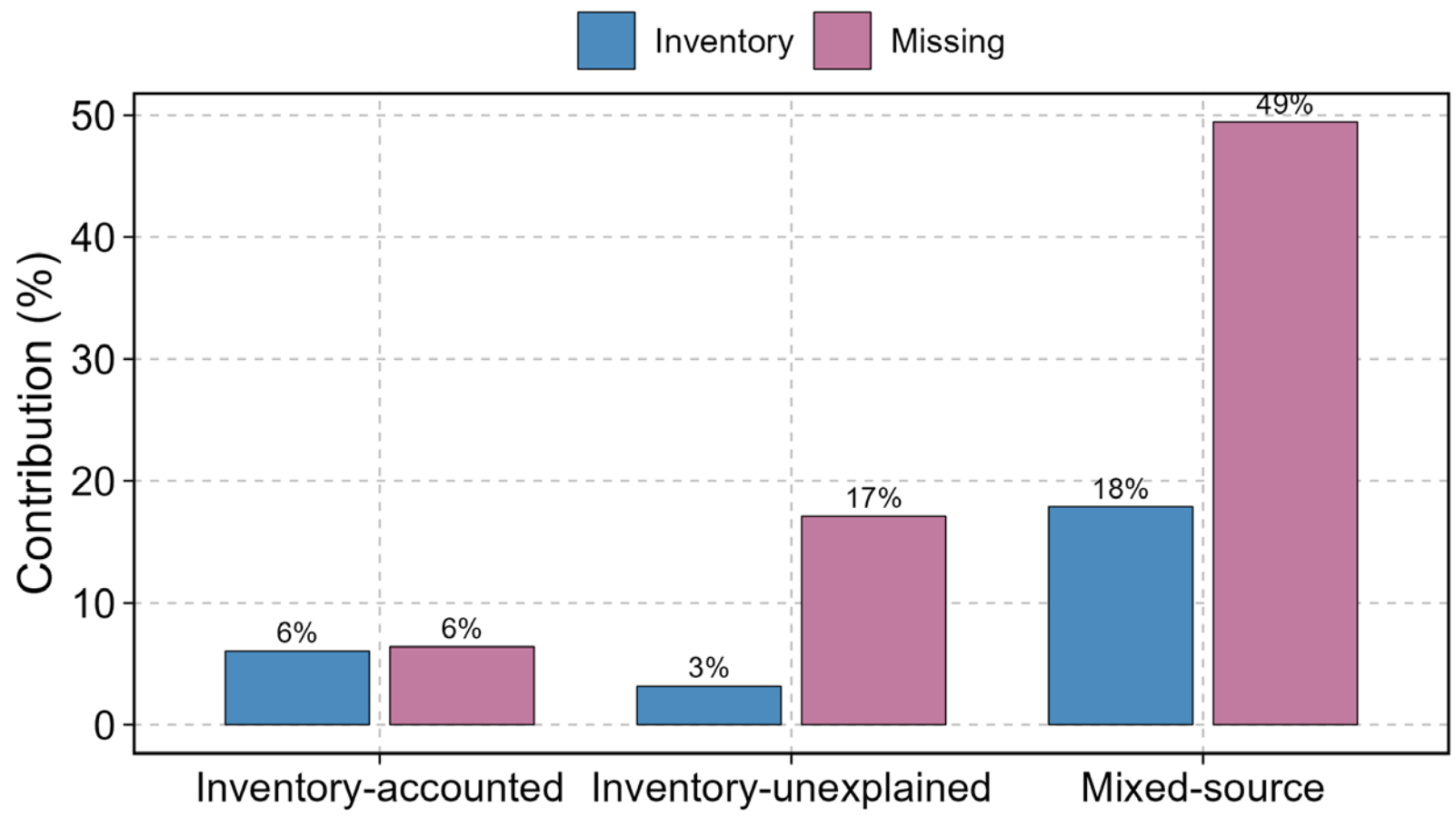

Supplementary Fig. 7. Breakdown of aircraft-derived monoterpenoid flux across the Los Angeles basin. Bars show the relative contributions of Inventory-accounted, Inventory-unexplained, and Mixed-source grid cells to the total observed flux, further separated into portions represented (Inventory) and unrepresented or underrepresented (Missing) in current emission inventories.

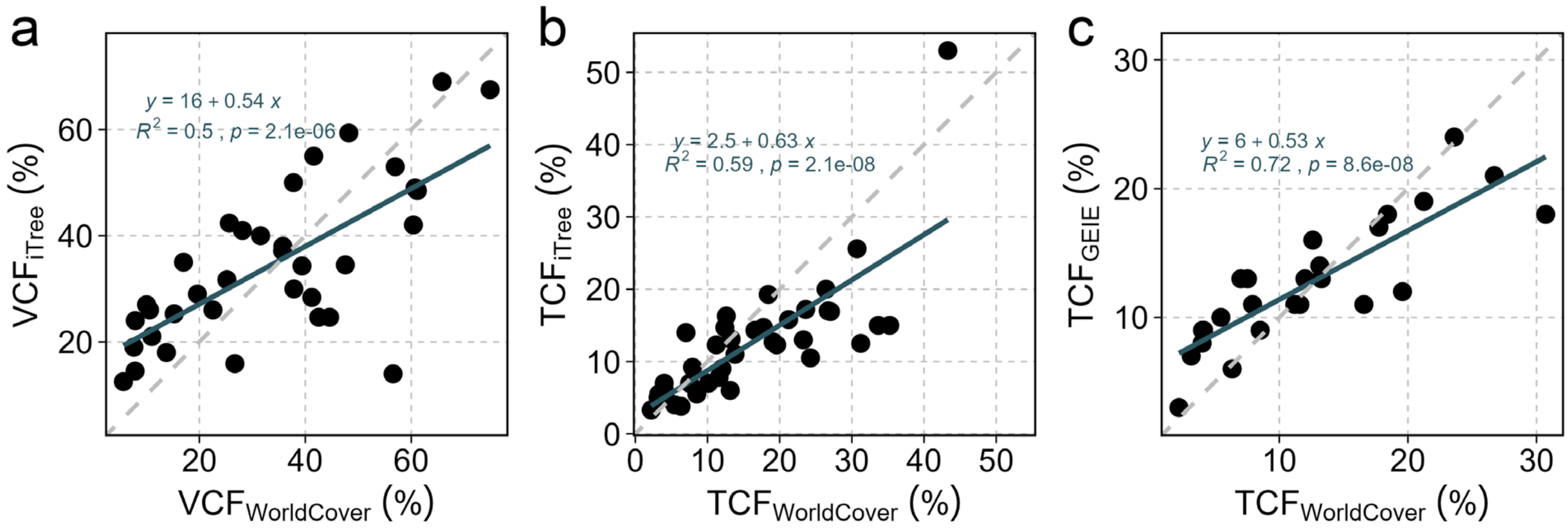

Supplementary Fig. 8. Comparison of city-level vegetation cover fraction (VCF) and tree cover fraction (TCF) estimates in major cities of California. a VCF from WorldCover compared with i-Tree estimates. **b** TCF from WorldCover compared with i-Tree estimates. **c** TCF from WorldCover compared with GEIE estimates. The green solid lines represent linear regressions, and the grey dashed lines denote 1:1 relationships.

Supplementary References

1. Pfannerstill, E. *et al.* Temperature-dependent emissions dominate aerosol and ozone formation in Los Angeles. *Science* **384**, 1324–1329 (2024).
2. Coggon, M. M. *et al.* Identifying and correcting interferences to PTR-ToF-MS measurements of isoprene and other urban volatile organic compounds. *Atmos. Meas. Tech.* **17**, 801–825 (2024).
3. Tani, A. Fragmentation and reaction rate constants of terpenoids determined by proton transfer reaction-mass spectrometry. *Environ. Control Biol.* **51,** 23–29 (2013).
4. Pagonis, D., Sekimoto, K. & De Gouw, J. A library of proton-transfer reactions of H_3O^+ ions used for trace gas detection. *J. Am. Soc. Mass Spectrom.* **30,** 1330–1335 (2019).
