# Supplementary material for: Reconciling observed and modeled estimates of urban terpenoid emissions
Source: Nat Commun. 2026 Jul 15;17:8671. doi: 10.1038/s41467-026-75075-9 (PMC13490599; doi:10.1038/s41467-026-75075-9)
Supplement: Supplementary file 2 [file 41467_2026_75075_MOESM2_ESM.pdf]

nature portfolio

Peer Review File

Reconciling observed and modeled estimates of urban terpenoid emissions

Corresponding Author: Dr Hui Wang

This file contains all reviewer reports in order by version, followed by all author rebuttals in order by version.

Attachments originally included by the reviewers as part of their assessment can be found at the end of this file.

Editorial Note: Parts of this Peer Review File have been redacted as indicated to remove third-party material where no permission to publish could be obtained.

Version 0:

Reviewer comments:

Reviewer #1

(Remarks to the Author)
Zhang et al present a valuable combination of airborne eddy-covariance VOC flux measurements together with mobile ground based concentration transects of isoprene and monoterpenoid compounds. They use these datasets to evaluate the performance of existing emission inventories as well as a modified version of the MEGAN model. Their analysis points to substantial unresolved anthropogenic sources of monoterpenoids that exhibit a temperature response, potentially resembling that of biogenic emissions, which they claim may lead to misclassification of these sources in current inventories. The optimised biogenic emission algorithm markedly improves the representation of isoprene fluxes; however, monoterpenoid emissions remain substantially underestimated, suggesting that temperature dependent emissions associated with industrial activity, food processing, florists, laundrettes, and car washes account for the unresolved emissions.

As societies transition toward low carbon economies, the relative importance of VOCs in shaping air quality is expected to grow, making the identification of unresolved emission sources increasingly important. These findings are therefore of clear interest to the atmospheric chemistry community, though I am less convinced that the broader relevance of the study has been fully demonstrated at this stage. While the analysis is well conducted, I don't think the main conclusions are fully supported by the available data and I elaborate below.

The discussion of isoprene appears briefly (lines 86–92), after which the manuscript concentrates almost exclusively on monoterpenoids, only returning to isoprene in the conclusions (lines 228–247). That final discussion, however, seems entirely based on speculation rather than clear interrogation of the presented data. As written, the modified MEGAN configuration substantially improves agreement between inventory and observations of isoprene — shifting from a strong inventory overestimation (especially for low fluxes, as evidenced by the intercept) to a remaining underestimation of 26%. This result is a real justification of the refined land cover approach and a real strength of the study. Yet the analysis of isoprene data stops there, and the authors do not explore the source of the remaining 26% discrepancy, instead, focusing solely on the much larger gaps for monoterpenoids. It is not obvious why an equally careful analysis is not applied to isoprene.

From Figures 1 and S1, my interpretation is that CARB_anthro and GRA2PESv1.1 appear to assume zero anthropogenic isoprene emissions. If correct, this is surprising, as several anthropogenic sources of isoprene are well-established – vehicle exhaust, combustion etc. The authors point out evidence for anthropogenic emissions of isoprene, particularly associated with food processing activities (143-145). With these contributions identified, why is the remaining isoprene underestimation discussed in terms of canopy microenvironment, emission factor inaccuracies and vegetation stress responses (lines 230–235)? The mobile transects show that isoprene is well correlated with monoterpenoids along multiple sections of the route (Fig. S5b). The monoterpenoids in those same sections are attributed to non inventory anthropogenic sources. Why, then, is isoprene not discussed similarly? The ratio of isoprene to monoterpenoid concentrations along those transects likely contains information about relative inventory biases. Without addressing potential anthropogenic contributions to isoprene, the discussion in the final paragraph is, in my view, insufficiently supported. Specifically, the sentence "we found that current inventories underestimate biogenic isoprene emissions by approximately 30%" is not true. The comparison was not between the inventory and total biogenic emissions, it was a comparison against total measured isoprene emissions which comprise

both biogenic and, as evidenced by the mobile transects, anthropogenic components.

A second concern is the claim that anthropogenic monoterpenoid emissions “exhibit a biogenic like temperature dependence, leading to systematic misclassification as biogenic”. While temperature dependencies are expected for all volatile organics, anthropogenic emissions also follow activity driven diurnal cycles, which typically help disentangle them from biogenic sources in well resolved time series. The airborne flux measurements in this study provide only a snapshot in space and time and therefore cannot reveal diurnal activity profiles in the way that fixed site ground based flux measurements (with stable footprints) could. In most cases, activity patterns are sufficient to separate anthropogenic and biogenic emissions, though potential exceptions may exist (e.g. VOC emissions from asphalt). For example, figure 4e does appear to show an exponential increase with temperature, but Figure 4f clearly shows a weekend reduction in anthropogenic monoterpenoids, implying that activity patterns do matter here. Figure 4d does not appear to show a temperature dependence consistent with biogenic emissions, as emissions are already decreasing at >30 C. For these reasons, I am not convinced that these emissions are ever likely to be “systematically misclassified” as biogenic and such a claim is currently overstated. Lines 208-210 suggest separating these factors into temperature-dependent categories – while this might be appropriate, there is an equally important need to better quantify their associated activity profiles.

My third point concerns the assignment of compounds to the ions measured by the PTR TOF instrument. Unless supported by GC MS or similar, PTR ion identifications are assumed rather than definitive, as multiple VOCs can produce the same product ions via fragmentation. It would therefore be appropriate for the Methods or Supplementary Information to include a discussion of the key ions reported, the compounds they are thought to represent, and the most likely sources of interference. For example, at m/z 69, typically attributed to isoprene, several studies have now demonstrated substantial contributions from fragmentation of anthropogenic VOCs, including higher carbon aldehydes and certain cycloalkanes. Recent work in urban environments suggests that these species can contribute up to ~50% of the m/z 69 signal during the daytime, with even larger interference at night (see manuscript ref. 40). Could there be similar interferences from anthropogenic sources contributing to the monoterpenoid ions?

Finally, some aromatic monoterpenes produce a significant fragment at m/z 93 e.g. p-cymene (E/N dependent) (see Tani et a., 2003 International Journal of Mass Spectrometry 223–224 (2003) 561–578), but would not have a parent fragment contribution to the monoterpene ions stated (e.g. m/z 137, 153, 155, 157). P-cymene has been linked to food preparation and cooking (e.g. Klein et al., 2016 - doi:10.1038/srep36623) and could conceivably be emitted from some of the implicated sources. With toluene central to the analysis, it would be important to discuss the potential for emissions of this monoterpene to act as an artefact to the toluene signal.

Minor comments
Lines 113-115. Elsewhere in the manuscript, octanal is mentioned as a tracer for cooking (line 160) and nonanal and acrolein are also associated with emissions from cooking (see manuscript ref 24). What does the correlation with relative bias look like for these tracers compared to the use of restaurant density?

Lines 80-97 I found this section difficult to follow and would recommend re-ordering to present the results of the old inventory approach first (Fig1a and 1b) and then move to describe the performance of the new approach (Fig1C and 1d). Presenting in that order seems more logical and would also mean figure panels are referred to in order of appearance.

Lines 80–97 – I am missing a clear, direct comparison between the standard MEGAN configuration (v3.2?) and the updated MEGAN version (v3.2_updated?). It is not clear whether the “standard” MEGAN used in this work corresponds to v3.2, or to the v3.0 referred to later on line 299. Am I correct in assuming that CARB_Bio essentially represents the standard MEGAN biogenic inventory? If so, then some of the most informative analysis would come from an explicit comparison of standard MEGAN (CARB_Bio) and MEGAN_updated against observations. Alternatively, if this is already what is shown by comparing panels 1a and 1c, given that CARB_anthro appears to include zero isoprene emissions, it would help if this were stated more explicitly. As noted above, the enhanced biogenic inventory is a real strength of this study, so clearly demonstrating how the updated MEGAN performs relative to the original is essential. Additionally, I would recommend restructuring the Materials and Methods so that the original MEGAN approach is introduced first, followed by the updated scheme, with a clear explanation of what has changed between the two.

Line 87 and throughout – Rather than mg/m2/h, I recommend using the SI standard: mg m-2 h-1

Line 274 – change to “…other stresses on BVOC emissions in this study” – its not clear what the other factors being introduced are. I suggest you end that sentence at “study”.

Line 321 I would include the E/N that the instrument was operated with as this will influence fragmentation (see point above).

Figs. 1, 2 and 4 Please change units from mg/m2/h to mg m-2 h-1

Figs S1, S2, S4 Please change units from mg/m2/h to mg m-2 h-1

Reviewer #2

(Remarks to the Author)
Attached

Version 1:

Reviewer comments:

Reviewer #1

(Remarks to the Author)
I have reviewed the revised manuscript and can see that the authors have carefully considered my comments and undertaken substantial additional analysis and revisions. I commend them for their thorough and thoughtful approach. I am satisfied that all of my concerns have been addressed and have no further comments. I am therefore happy to recommend this manuscript for publication.

Open Access This Peer Review File is licensed under a Creative Commons Attribution 4.0 International License, which permits use, sharing, adaptation, distribution and reproduction in any medium or format, as long as you give appropriate credit to the original author(s) and the source, provide a link to the Creative Commons license, and indicate if changes were made.
In cases where reviewers are anonymous, credit should be given to 'Anonymous Referee' and the source.
The images or other third party material in this Peer Review File are included in the article's Creative Commons license, unless indicated otherwise in a credit line to the material. If material is not included in the article's Creative Commons license and your intended use is not permitted by statutory regulation or exceeds the permitted use, you will need to obtain permission directly from the copyright holder.
To view a copy of this license, visit https://creativecommons.org/licenses/by/4.0/

Response to Reviewers' Comments

The reviewer's comments are listed below in *black italics*, and our responses and changes in the manuscript are highlighted in blue and red, respectively.

Response to Reviewer #1:

Zhang et al present a valuable combination of airborne eddy-covariance VOC flux measurements together with mobile ground based concentration transects of isoprene and monoterpenoid compounds. They use these datasets to evaluate the performance of existing emission inventories as well as a modified version of the MEGAN model. Their analysis points to substantial unresolved anthropogenic sources of monoterpenoids that exhibit a temperature response, potentially resembling that of biogenic emissions, which they claim may lead to misclassification of these sources in current inventories. The optimised biogenic emission algorithm markedly improves the representation of isoprene fluxes; however, monoterpenoid emissions remain substantially underestimated, suggesting that temperature dependent emissions associated with industrial activity, food processing, florists, laundrettes, and car washes account for the unresolved emissions.

As societies transition toward low carbon economies, the relative importance of VOCs in shaping air quality is expected to grow, making the identification of unresolved emission sources increasingly important. These findings are therefore of clear interest to the atmospheric chemistry community, though I am less convinced that the broader relevance of the study has been fully demonstrated at this stage. While the analysis is well conducted, I don't think the main conclusions are fully supported by the available data and I elaborate below.

Response: We thank the reviewer for the careful evaluation of our manuscript and are encouraged that the study is considered of clear interest to the atmospheric chemistry community. We also thank the reviewer for raising the important concern regarding the broader relevance and support for our main conclusions. In response, we have revised the manuscript in several key aspects, as summarized below.

i) We have expanded the analysis of isoprene to provide a more balanced and detailed evaluation alongside monoterpenoids.

ii) We have added a more detailed description of the VOC measurements using Vocus PTR-ToF-MS during airborne flux observations.

iii) We have clarified the broader relevance of our findings by highlighting that the key implication of this study is not limited to the Los Angeles region, but reflects a more general limitation in current emission inventories.

We believe these revisions better align the conclusions with the available evidence, while still conveying the significance of the identified gap in current emission inventories.

1. The discussion of isoprene appears briefly (lines 86–92), after which the manuscript concentrates almost exclusively on monoterpenoids, only returning to isoprene in the conclusions (lines 228–247). That final discussion, however, seems entirely based on speculation rather than clear interrogation of the presented data. As written, the modified MEGAN configuration substantially improves agreement between inventory and observations of isoprene — shifting from a strong inventory overestimation (especially for low fluxes, as evidenced by the intercept) to a remaining underestimation of 26%. This result is a real justification of the refined land cover approach and a real strength of the study. Yet the analysis of isoprene data stops there, and the authors do not explore the source of the remaining 26% discrepancy, instead, focusing solely on the much larger gaps for monoterpenoids. It is not obvious why an equally careful analysis is not applied to isoprene.

From Figures 1 and S1, my interpretation is that CARB_anthro and GRA2PESv1.1 appear to assume zero anthropogenic isoprene emissions. If correct, this is surprising, as several anthropogenic sources of isoprene are well-established – vehicle exhaust, combustion etc. The authors point out evidence for anthropogenic emissions of isoprene, particularly associated with food processing activities (143-145). With these contributions identified, why is the remaining isoprene underestimation discussed in terms of canopy microenvironment, emission factor inaccuracies and vegetation stress responses (lines 230–235)? The mobile transects show that isoprene is well correlated with monoterpenoids along multiple sections of the route (Fig. S5b). The monoterpenoids in those same sections are attributed to non inventory anthropogenic sources. Why, then, is isoprene not discussed similarly? The ratio of isoprene to monoterpenoid concentrations along those transects likely contains information about relative inventory biases. Without addressing potential anthropogenic contributions to isoprene, the discussion in the final paragraph is, in my view, insufficiently supported. Specifically, the sentence "we found that current inventories underestimate biogenic isoprene emissions by approximately 30%" is not true. The comparison was not between the inventory and total biogenic emissions, it was a comparison against total measured isoprene emissions which comprise both biogenic and, as evidenced by the mobile transects, anthropogenic components.

Response: We thank the reviewer for this careful and constructive comment. We agree that, in the original manuscript, the discussion of isoprene was comparatively brief and that the remaining discrepancy between inventory estimates and airborne measurements was not examined with the same level of detail as for monoterpenoids. We also agree that our original concluding discussion placed too much emphasis on interpretation without presenting sufficient analysis in the main text to support those statements. We have therefore revised the manuscript by expanding the isoprene analysis, clarifying the role of anthropogenic contributions, and moderating our interpretation of the residual inventory–measurement difference.

First, we would like to clarify that anthropogenic emissions of isoprene are not zero in the $CARB_{Anthro}$ and $GRA^2PESv1.1$ inventories used in this study. However, they are represented as small relative to biogenic sources and therefore contribute negligibly to total isoprene emissions

at the grid cell scale considered here. Accordingly, the inventories indicate that biogenic sources dominate isoprene emissions regionally. In addition, our airborne flux measurements showed that high isoprene emissions were primarily associated with vegetated and remote areas (revised Fig. 1e), while fluxes remain relatively low in urban cores, supporting the dominance of biogenic isoprene sources at the regional scale.

At the same time, we acknowledge that anthropogenic sources of isoprene may contribute locally and could be underrepresented in current inventories. However, we do not interpret the remaining 26% inventory–measurement difference as direct evidence for a systematic underestimate of anthropogenic isoprene emissions at the regional scale. This residual difference is relatively modest and is within the uncertainty range of airborne flux measurements[1]. Therefore, the residual difference should be interpreted cautiously.

To assess whether the remaining differences showed any systematic source-related patterns, we have now conducted the same level of analysis for isoprene as for monoterpenoids. We examined correlations between isoprene relative bias and tracer of various activities (revised Figs. 2a, S2). Significant negative correlations were found with acetonitrile ($r = –0.22$, $p < 0.05$) and naphthalene ($r = –0.21$, $p < 0.05$), suggesting that part of the residual discrepancy may be associated with incompletely represented combustion-related anthropogenic influences. Spatial patterns of isoprene relative bias further showed larger negative biases in Long Beach and Colton (revised Fig. 1f), areas characterized by intense industrial and traffic activities. These tracers and spatial patterns do not allow a unique attribution to specific combustion sectors, and we therefore avoid assigning the residual discrepancy specifically to vehicle exhaust emissions. Instead, the results indicate that larger residual discrepancies occurred in mixed urban industrial environments, where combustion-related anthropogenic influences may contribute locally. This interpretation is consistent with previous observations in Houston showing anthropogenic isoprene enhancements associated with industrial areas[2,3].

To further quantify the influence of anthropogenic contributions, we categorized grid cells as "Biogenic-dominated" and "Mixed-source" to refine the evaluations of isoprene emissions (Fig. S6). In grid cells dominated by biogenic sources (defined as TCF ≥ 5% and acetonitrile flux ≤ 0.01 mg m^{-2} h^{-1}; 52% of all grid cells), the inventory explained 88% of observed isoprene fluxes (R^2 = 0.41). In contrast, in grid cells influenced by both biogenic and anthropogenic sources (remaining 48%), the explained fraction decreased to 55% (R^2 = 0.30). This comparison indicates that residual differences were more evident in mixed-source environments where anthropogenic contributions were present, whereas biogenic-dominated regions were well captured by the inventory. Importantly, because a large fraction of total isoprene emissions originated from biogenic-dominated grid cells, this localized discrepancies had a limited impact on the overall isoprene emissions.

Accordingly, we have revised the manuscript to expand the analysis of isoprene, as detailed below. Revised Figs. 1 and 2 have replaced the original Figs. 1 and 2 in the manuscript.

Lines 93–112: "We then evaluated the updated inventory developed in this study, which combines MEGANv3.2$_{Updated}$ with GRA^{2}PESv1.1 (Figs. 1c–d). Within this framework, biogenic sources accounted for nearly all isoprene emissions. Consistent with this, airborne flux measurements showed that high isoprene emissions were primarily associated with vegetated and remote areas (Fig. 1e), supporting the dominance of biogenic sources at the regional scale. The updated inventory reasonably captured the spatial distribution of isoprene ($R^2 = 0.37$) and explained 74% (0.40 ± 0.33 mg m^{-2} h^{-1}) of observed values, showing a substantial improvement compared with previous inventories (Fig. 1a). Similar results were derived when GRA^{2}PESv1.1 was replaced with another commonly used anthropogenic inventory, CARB$_{Anthro}$ (Fig. S1), showing that the improvement arises mainly from refined biogenic inputs of urban trees and landcover maps. The remaining 26% inventory–measurement difference is relatively modest and within the uncertainty range of airborne flux measurements[14]. Nevertheless, we further examined whether the residual differences exhibited systematic source-related patterns (Figs. 1f, 2a, S2). Larger negative biases were observed in several urban industrial areas, including Long Beach and Colton (yellow squares in Fig. 1f). In addition, isoprene relative bias showed significant negative correlations with acetonitrile ($r = -0.22$, $p < 0.05$) and naphthalene ($r = -0.21$, $p < 0.05$), tracers associated with combustion-related and mixed anthropogenic influences (Fig. 2a). These results suggest that unresolved urban anthropogenic influences may contribute locally to the residual discrepancy. This interpretation is consistent with previous gas chromatography (GC)-based observations in Houston showing anthropogenic isoprene enhancements in industrialized urban environments[23,24].

Lines 198–211: "Isoprene and monoterpenoid emissions were influenced by different sources, so we applied source-specific indicators to categorize grid cells and reconcile observed and modeled fluxes. Our above analyses indicated that isoprene emissions were regionally dominated by biogenic sources, with possible additional local contributions from industrial anthropogenic activities, whereas monoterpenoid emissions arose from a broader mix of biogenic and multiple anthropogenic sources, including food industry and commercial activities. Therefore, different tracer-based criteria were used to classify grid cells for isoprene and monoterpenoids, respectively.

For isoprene, we categorized grid cells as "Biogenic-dominated" and "Mixed-source" (Fig. S6). In grid cells dominated by biogenic sources (defined as TCF $\geq$ 5% and acetonitrile flux ≤ 0.01 mg m^{-2} h^{-1} in Fig. 2c; 52% of all grid cells), the inventory explained 88% of observed isoprene fluxes ($R^2 = 0.41$). In contrast, in mixed-source grid cells (remaining 48%), the explained fraction decreased to 55% ($R^2 = 0.30$). This comparison indicates that residual discrepancies were more pronounced in mixed-source environments, where local anthropogenic influences may be less well represented by current inventories."

Lines 273–279: "Our results also provide insights into refining urban biogenic emission models. The substantial improvement in isoprene estimates after updating urban vegetation inputs demonstrates the importance of accurately representing urban land cover and vegetation composition. However, residual inventory–measurement differences remain, especially for monoterpenoids. Although these differences cannot be attributed solely to biogenic emissions, they

highlight remaining uncertainties in urban BVOC modeling, including canopy microenvironment, emission factors, and missing processes such as vegetation stress responses and non-foliar emissions……"

[Redacted]

Revised Fig. 1. Evaluation of terpenoid emissions against airborne flux observations. a,b,c,d, Scatterplots comparing inventory estimates with aircraft flux observations for **(a, c)** isoprene and **(b, d)** monoterpenoids. For **a** and **b**, light purple points and regression lines indicate the combined MEGANv3.2$_{Default}$ + GRA^{2}PESv1.1 inventories, while blue points and regression lines indicate

the combined $CARB_{Bio}$ + $CARB_{Anthro}$ inventories, reproduced following Pfannerstill et al. (2024)[14]. For **c** and **d**, purple points and regression lines indicate the combined $MEGANv3.2_{Updated}$ + $GRA^2PESv1.1$ inventories, while green points and regression lines represent the $MEGANv3.2_{Updated}$ biogenic inventory. In **c**, the two regressions largely overlap, reflecting the minor contribution of anthropogenic emissions to isoprene and the dominance of biogenic sources. **(e, g)** Spatial distribution of aircraft-derived isoprene and monoterpenoid flux. The yellow square highlights the Vernon and Commerce area. **(f, h)** Relative bias between inventory and airborne observations, calculated as (Inventory − Observation)/Observation. The yellow squares highlight the Colton and Long Beach area.

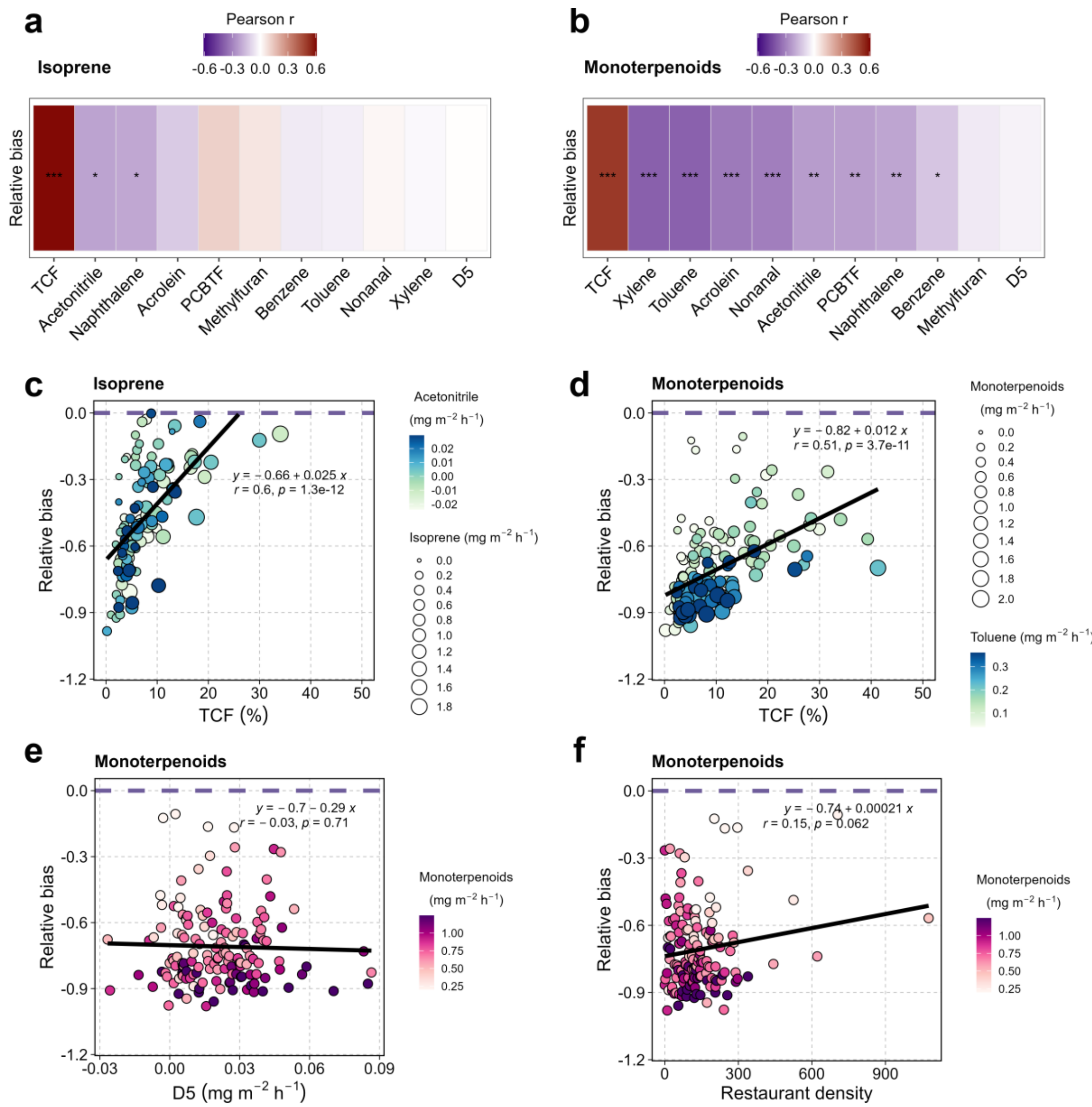

Revised Fig. 2. Relationship between relative bias of the combined $MEGANv3.2_{Updated}$ + $GRA^2PESv1.1$ terpenoid inventories and various emission indicators. a, b, Pearson

correlations between relative bias in **(a)** isoprene and **(b)** monoterpenoid fluxes and tree cover fraction (TCF, %) and airborne fluxes of selected chemical species. Colors indicate pearson correlation coefficients (r), and asterisks denote statistical significance (*$p < 0.1$; **$p < 0.01$; ***$p < 0.001$). **c, d,** Isoprene relative bias and monoterpenoid relative bias versus TCF, colored by (**c**) acetonitrile flux and (**d**) toluene flux. Marker size denotes aircraft-derived isoprene and monoterpenoid flux, and marker color indicates the co-located emission proxy in each panel. **e, f,** Monoterpenoid relative bias versus anthropogenic indicators, including (**e**) D5 flux and (**f**) restaurant density. Restaurant locations in Los Angeles were obtained via the Google Maps API, and grid-cell counts were used as a proxy for cooking intensity. Solid black lines represent least-squares fits, with r and p-values indicated.

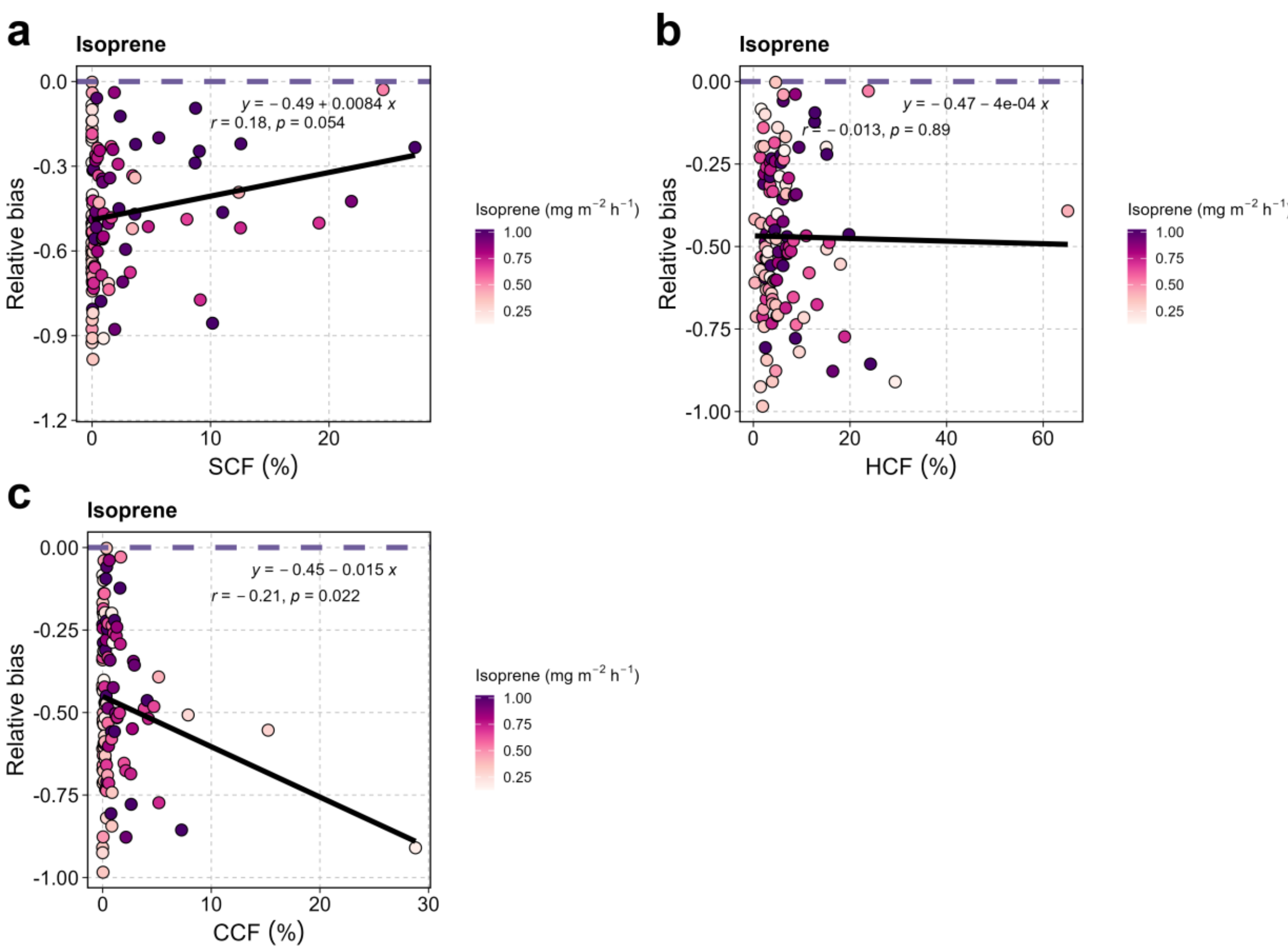

Fig. S2. Relationship between relative bias in isoprene flux and non-tree biogenic sources. a, shrub cover fraction (SCF, %). **b,** herbaceous cover fraction (HCF, %). **c,** crop cover fraction (CCF, %). Marker color denotes aircraft-derived isoprene flux. Solid black lines indicate least-squares fits, with Pearson r and p values provided.

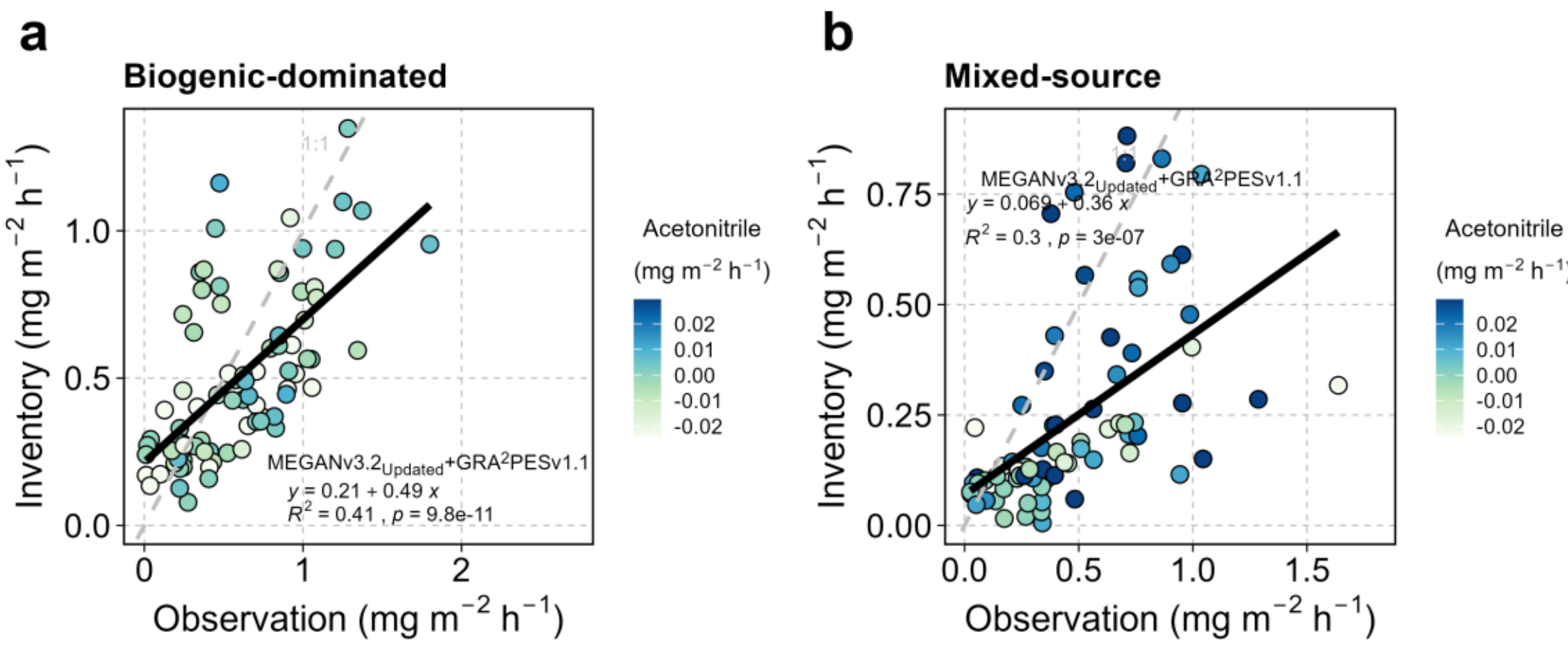

Fig. S6. Comparison of isoprene emissions in Biogenic-dominated and Mixed-source grid cells. Scatterplots of inventory estimates ($MEGANv3.2_{Updated}$ + $GRA^2PESv1.1$) against airborne flux observations for (**a**) Biogenic-dominated (acetonitrile flux ≤ 0.01 mg m^{-2} h^{-1}, TCF $\geq 5\%$), and (**b**) Mixed-source grid cells.

2. A second concern is the claim that anthropogenic monoterpenoid emissions "exhibit a biogenic like temperature dependence, leading to systematic misclassification as biogenic". While temperature dependencies are expected for all volatile organics, anthropogenic emissions also follow activity driven diurnal cycles, which typically help disentangle them from biogenic sources in well resolved time series. The airborne flux measurements in this study provide only a snapshot in space and time and therefore cannot reveal diurnal activity profiles in the way that fixed site ground based flux measurements (with stable footprints) could. In most cases, activity patterns are sufficient to separate anthropogenic and biogenic emissions, though potential exceptions may exist (e.g. VOC emissions from asphalt). For example, figure 4e does appear to show an exponential increase with temperature, but Figure 4f clearly shows a weekend reduction in anthropogenic monoterpenoids, implying that activity patterns do matter here. Figure 4d does not appear to show a temperature dependence consistent with biogenic emissions, as emissions are already decreasing at >30 C. For these reasons, I am not convinced that these emissions are ever likely to be "systematically misclassified" as biogenic and such a claim is currently overstated.

Response: We thank the reviewer for this thoughtful comment and agree that our original wording was somewhat too strong. In particular, we agree that the phrase "systematically misclassified as biogenic" was more definitive than can be supported by the available evidence. We also agree that activity-driven temporal variability is important for distinguishing anthropogenic from biogenic emissions. While fixed-site measurements are better suited to resolving detailed diurnal patterns at a given location, our objective here is to understand source patterns at the scale of the Los Angeles region. In that context, airborne flux measurements provide valuable regional scale constraints on the spatial distribution of emissions across the basin, although they do not fully capture activity-driven temporal patterns in the same way as continuous fixed-site observations. We appreciate this point and have revised the manuscript accordingly.

Our intention was not to suggest that anthropogenic monoterpenoid emissions are indistinguishable from biogenic sources, but rather to highlight that anthropogenic sources can exhibit strong temperature-dependent emissions that may resemble biogenic emission patterns under certain conditions. For example, Fig. 4e shows an exponential increase in flux with temperature in inventory-unexplained grid cells, which are dominated by anthropogenic emissions. The strong temperature dependence observed in anthropogenic-dominated grid cells demonstrates that anthropogenic sources can show temperature responses comparable to those typically associated with biogenic emissions. At the same time, Fig. 4f shows clear weekday–weekend differences, indicating that both temperature and activity patterns influence the observed emissions.

Taken together, these results indicate that it would be important to consider temperature-dependence for future anthropogenic emission inventory development. In most current emission inventories, anthropogenic emissions are treated as temperature-independent, while biogenic emissions are parameterized as strongly temperature-dependent. This difference in representation may bias model-based source attribution under high-temperature conditions, because temperature-driven increases in anthropogenic emissions could be attributed to biogenic sources when anthropogenic emissions are assumed to be temperature-independent within the inventory framework.

To avoid overstating this point, we have removed the statement that anthropogenic emissions are "systematically misclassified as biogenic" and revised the manuscript to better reflect both the role of temperature sensitivity and the importance of activity patterns. The revised descriptions are as follows.

Lines 25–27: "We identify that these substantial emissions originate from the previously overlooked food industry and small commercial businesses (e.g., florists, car washes, and laundries), which exhibit a biogenic-like temperature dependence~~, leading to their systematic misclassification as biogenic~~."

Lines 238–245: "Similar to the Inventory-accounted category, the Inventory-unexplained category also exhibited a strong temperature dependence (Figs. 4d–e), effectively mimicking the emission behavior of vegetation. However, the anthropogenic nature of these sources is clearly confirmed by the marked weekday–weekend variations in emission intensity (Fig. 4f), a temporal signature absent in biogenic emissions. These results highlight the need to explicitly incorporate both the temporal variability and temperature dependence of these sources into current emission inventories and air quality management frameworks."

Lines 249–266: "……By integrating grid-cell information from refined inventories with airborne flux and ground-based mobile measurements, we identified distinct additional anthropogenic sources from the food industry and small commercial activities, such as florists, laundry, and car wash facilities. <u>These emissions appear locally intense and exhibit temperature sensitivity that may resemble biogenic behavior under certain conditions, potentially complicating source attribution if not properly represented in emission inventories.</u>

Understanding the sources, activity profile, and temperature sensitivity of terpenoid emissions offers guidance for urban air quality management. While recent studies have highlighted the climate penalty of temperature-driven anthropogenic emissions[13], our findings point to a broader limitation in current emission frameworks in that temperature-dependent processes in certain anthropogenic sectors may not be adequately captured. This limitation may also extend to other plausible sources, such as asphalt-related emissions from road pavement, which have been shown to increase with temperature and could contribute to urban air pollution[31,32]. Re-attributing these hidden anthropogenic emissions and incorporating both activity-related temporal variability and temperature dependence into emission inventories may improve the representation of anthropogenic VOC emissions. In particular, distinguishing between temperature-independent, temperature-dependent, and strongly temperature-dependent anthropogenic emissions could help improve the representation of urban VOC emissions in chemical transport models under a changing climate."

Lines 208-210 suggest separating these factors into temperature-dependent categories – while this might be appropriate, there is an equally important need to better quantify their associated activity profiles.

Response: We agree that activity-driven temporal patterns are an important characteristic of anthropogenic emissions and should be better quantified in emission inventories. Some existing inventories already included activity-based temporal allocation. For example, the FIVE-VCP inventory applied different temporal profiles for weekdays, Saturdays, and Sundays, as well as diurnal variation for several source categories. However, despite these advances, most anthropogenic emission inventories still lack explicit parameterizations of temperature-dependent emission processes. Our airborne flux observations, together with recent studies[4,5], suggest that some anthropogenic sources exhibit temperature sensitivity that is not fully represented in current inventories.

Therefore, the intent of Lines 208–210 in the original manuscript was to suggest that temperature dependence represents an additional dimension that is often missing from current anthropogenic VOC emission frameworks in addition to activity patterns. Classifying anthropogenic VOC emissions into temperature-independent, temperature-dependent, and strongly temperature-dependent categories could help improve emission modeling under changing climate conditions, while complementary improvements in activity-based temporal profiles will also be needed.

We have clarified this point in the manuscript and highlighted the need to better represent both activity-related temporal variations and temperature dependence in future inventories, see revised text above (**Lines 255–266**).

3. My third point concerns the assignment of compounds to the ions measured by the PTR TOF instrument. Unless supported by GC MS or similar, PTR ion identifications are assumed rather than definitive, as multiple VOCs can produce the same product ions via fragmentation. It would therefore be appropriate for the Methods or Supplementary Information to include a discussion of

the key ions reported, the compounds they are thought to represent, and the most likely sources of interference. For example, at m/z 69, typically attributed to isoprene, several studies have now demonstrated substantial contributions from fragmentation of anthropogenic VOCs, including higher carbon aldehydes and certain cycloalkanes. Recent work in urban environments suggests that these species can contribute up to ~50% of the m/z 69 signal during the daytime, with even larger interference at night (see manuscript ref. 40). Could there be similar interferences from anthropogenic sources contributing to the monoterpenoid ions?

Response: We agree that PTR-ToF-MS ion assignments are not inherently definitive, as multiple VOCs can contribute to the same product ions via fragmentation. In the revised manuscript, we have now explicitly summarized the key ions used in this study, their likely assignments, and known interferences, together with the relevant instrument operating conditions. We have further clarified that the airborne flux dataset used here has been described in detail in Pfannerstill et al. (2024)[1], including the PTR-ToF-MS calibration procedures and the correction approaches applied to account for known interferences. In addition, as an independent line of support, we compared PTR-ToF-MS measurements from the NOAA mobile-laboratory with GC-based measurements in the same Los Angeles region, which showed good agreement for key compounds such as isoprene (Fig. R1). These additions provide a clearer basis for evaluating both the uncertainty and the robustness of the ion assignments used in this work.

During the RECAP-CA flights, the Vocus PTR-ToF-MS was operated at a reactor temperature of 60 °C, pressure of 2.0 mbar, and E/N of ca. 130 Td, with the skimmer 1–2 voltage difference varying from 6 to 9.1 V during the campaign, which affects fragmentation and sensitivity, and these effects were accounted for through calibration and interference corrections[1].

For m/z 69 ($C5H8H^+$), interference from anthropogenic VOCs such as cycloalkanes and long-chain aldehydes were explicitly accounted for, as detailed in Pfannerstill et al. (2024)[1]. The correction approach of Coggon et al. (2024)[6] was applied, in which m/z 69.0699 is adjusted based on its relationship with fragment ions m/z 111.117 and 125.132 derived from nonanal calibration and observations over oil and gas fields in the San Joaquin Valley. While this correction affects mixing ratios in the urban background, its impact on fluxes is small (10%), indicating that most of the covariance signal reflects true isoprene emissions.

As additional supporting evidence, we compared isoprene concentrations measured by PTR-ToF-MS with those measured by GC-MS during NOAA mobile-laboratory observations in Los Angeles (Fig. R1). The two measurements were well correlated (R^2 = 0.81; slope = 0.97), indicating that the PTR-ToF-MS signal tracked the spatial variability of isoprene well across the sampled region. We also note that PTR-ToF-MS showed a positive intercept relative to GC-MS (0.33 ppbv), and some individual samples were higher in PTR-ToF-MS, suggesting a residual background contribution and/or partial interference from other compounds. However, the close overall relationship indicates that isoprene remained the dominant contributor to the PTR-ToF-MS signal at the regional scale. This comparison does not exclude interference entirely, but it supports the

interpretation that, in the Los Angeles region, the m/z 69 signal primarily reflects isoprene with relatively little interference from other compounds.

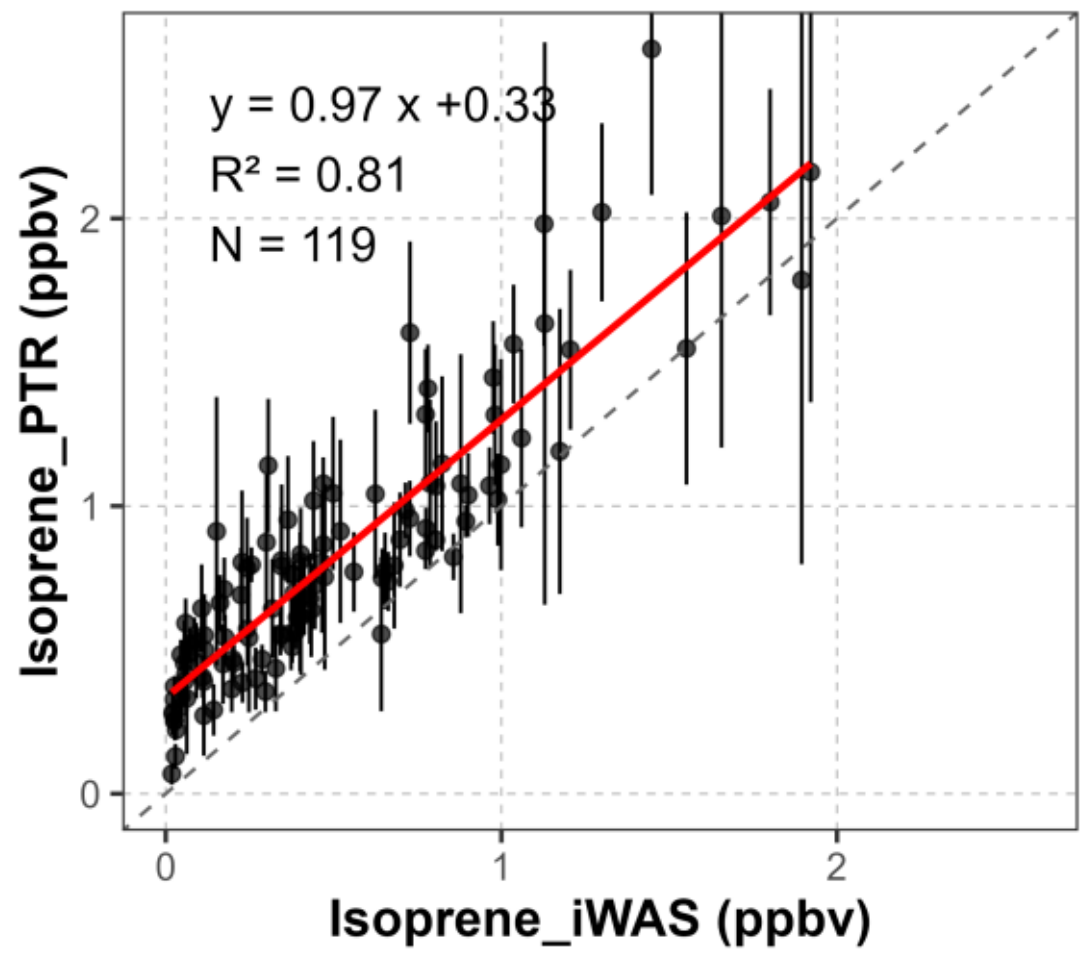

Fig. R1. Comparison of isoprene concentrations measured by PTR-ToF-MS and by GC-MS during NOAA mobile-laboratory observations in Los Angeles. Each point represents measurements averaged over the GC-MS sampling interval. Error bars denote the standard deviation of PTR-ToF-MS measurements within the corresponding averaging period. The red line indicates the least-squares regression, and the dashed line represents the 1:1 relationship.

For the m/z 137.13 ion ($C10H16H^+$), potential contributing species were also evaluated in Pfannerstill et al. (2024)[1]. This ion is referred to here as a monoterpenoid signal because it can include contributions from both non-functionalized monoterpenes and fragments from oxygenated monoterpenoids or monoterpene alcohols with parent mass m/z 155.14 (e.g., eucalyptol, linalool, cineole, terpineol)[7]. However, interference from sesquiterpene fragmentation is expected to be negligible, as only 5% of sesquiterpenes fragment to m/z 137.13 and their concentrations and fluxes are about an order of magnitude lower than monoterpenes, implying an interference below 1%. In addition, VOCs shown individually in the manuscript figures, contributing substantially to the fluxes, or driving inventory discrepancies were gas-standard calibrated and/or have well-characterized fragmentation behavior, such that remaining interferences are expected to be small.

As additional supporting evidence, we compared the PTR-ToF-MS m/z 137.13 monoterpenoid signal with speciated monoterpene concentrations measured by GC-MS during NOAA mobile-laboratory observations in Los Angeles (Fig. R2). For the GC-MS measurements, only α-pinene and β-pinene were available from the NOAA SUNVEx dataset, so their sum was used for this comparison. One outlier point was located near specific gas station (Fig. R2a) and car wash point sources and may have been influenced by other speciated monoterpenes or oxygenated monoterpenoids that were not accessible from the public GC-MS dataset. After excluding measurements collected near the specific point source and focusing on transect data to evaluate consistency between the two methods, the PTR-ToF-MS and GC-MS measurements were well

correlated (R^2 = 0.74; slope = 0.89; Fig. R2b), indicating that the PTR-ToF-MS m/z 137.13 signal tracked the spatial variability of monoterpenes across the sampled region. We note that PTR-ToF-MS showed a small positive intercept relative to GC-MS (0.05 ppbv), and some individual samples were higher in PTR-ToF-MS, suggesting a residual background contribution and/or contributions from additional monoterpenoid species or fragments. This comparison does not exclude such contributions entirely, but it supports the interpretation that the PTR-ToF-MS m/z 137.13 signal provides a reasonable regional scale proxy for monoterpenoid variability in the Los Angeles region.

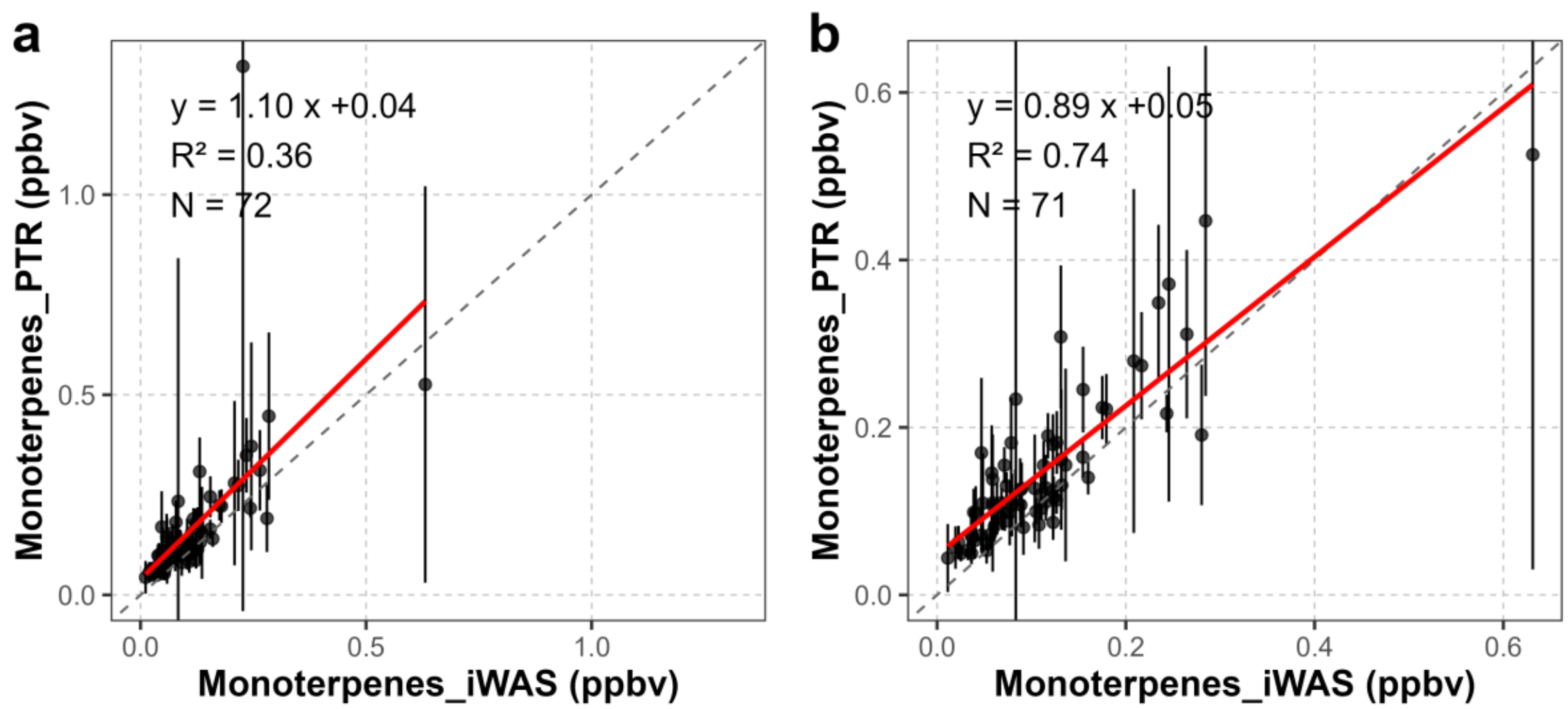

Fig. R2. Comparison of monoterpene concentrations measured by PTR-ToF-MS and by GC-MS during NOAA mobile-laboratory observations in Los Angeles. Each point represents measurements averaged over the GC-MS sampling interval. Error bars denote the standard deviation of PTR-ToF-MS measurements within the corresponding averaging period. The red line indicates the least-squares regression, and the dashed line represents the 1:1 relationship.

We have added the following contents in the revised Methods and SI summarizing key ions, likely compound assignments, potential interferences, and applied corrections.

Lines 370–374: "Airborne flux measurement. Flux measurements were derived using airborne eddy covariance onboard a Twin Otter aircraft equipped with a Vocus PTR-ToF-MS (Proton Transfer Reaction Time of Flight Mass Spectrometer, Aerodyne Inc., Billerica, MA, USA) for VOCs during June 2021[14]. During the flights, the PTR-ToF-MS was operated at a reactor temperature of 60 °C, pressure of 2.0 mbar, and E/N of ca. 130 Td. Key ions, likely compound assignments, and potential interferences for the PTR-ToF-MS measurements are provided in Text S1……"

SI: "Text S1. Key ions and potential sources of interference from Vocus PTR-ToF-MS measurements during airborne flux measurements. For m/z 69 ($C5H8H^+$), interference from anthropogenic VOCs such as cycloalkanes and long-chain aldehydes were accounted for explicitly[1]. The correction approach of Coggon et al. (2024)[2] was applied, in which m/z 69.0699

was adjusted based on its relationship with fragment ions m/z 111.117 and 125.132 derived from nonanal calibration and observations over oil and gas fields in the San Joaquin Valley[1]. While this correction affected mixing ratios in the urban background, its impact on fluxes was small (10%), indicating that most of the covariance signal reflects true isoprene emissions.

For the monoterpene ion (m/z 137.13), potential contributing species were also evaluated[1]. This ion is referred to here as a monoterpenoid signal because it can include contributions from both non-functionalized monoterpenes and fragments from oxygenated monoterpenoids or monoterpene alcohols with parent mass m/z 155.14 (e.g., eucalyptol, linalool, cineole, terpineol)[3]. However, interference from sesquiterpene fragmentation is expected to be negligible, as only 5% of sesquiterpenes fragment to m/z 137.13 and their concentrations and fluxes were about an order of magnitude lower than monoterpenes, implying an interference below 1%[1].

In addition, VOCs shown individually in the figures, e.g., isoprene, monoterpenes (mixture of α- and β-pinene and limonene), acetonitrile, acrolein, benzene, toluene, xylene, D5 siloxane, nonanal, and furan, contributing substantially to the fluxes, or driving inventory discrepancies were gas-standard calibrated and/or have well-characterized fragmentation behavior[2,4], such that remaining interferences are expected to be small."

4. Finally, some aromatic monoterpenes produce a significant fragment at m/z 93 e.g. p-cymene (E/N dependent) (see Tani et a., 2003 International Journal of Mass Spectrometry 223–224 (2003) 561–578), but would not have a parent fragment contribution to the monoterpene ions stated (e.g. m/z 137, 153, 155, 157). P-cymene has been linked to food preparation and cooking (e.g. Klein et al., 2016 - doi:10.1038/srep36623) and could conceivably be emitted from some of the implicated sources. With toluene central to the analysis, it would be important to discuss the potential for emissions of this monoterpene to act as an artefact to the toluene signal.

Response: We thank the reviewer for raising this important point regarding potential contributions of aromatic monoterpenes such as p-cymene to the m/z 93 signal. We agree that certain aromatic monoterpenes can produce fragments at m/z 93 under PTR-ToF-MS conditions, and that this fragmentation is dependent on E/N[7]. During the RECAP-CA flights, the Vocus PTR-ToF-MS was operated at E/N of ca. 130 Td[1], under which such fragmentation pathways cannot be entirely excluded. We have therefore added discussion of this potential interference in the revised manuscript.

However, we do not expect this potential contribution to alter the main interpretation of our analysis. Our interpretation of the monoterpenoid relative bias does not rely solely on toluene or the m/z 93 ion assignment, but is supported by consistent relationships across multiple tracers and independent emission indicators. In addition to toluene, other aromatic VOCs such as xylene also exhibit significant negative correlations with monoterpenoid relative bias (e.g., $r = -0.39$, $p < 0.001$), together with oxygenated VOCs associated with lipid oxidation or thermal processes (e.g., acrolein and nonanal). These consistent multi-species relationships indicate a broader pattern linked to anthropogenic activities (e.g., solvent use and food-related processes), rather than a single

compound-specific artifact. In addition, even if p-cymene contributes partly to m/z 93 in some locations, it may still be associated with anthropogenic source types relevant to our interpretation, such as food-related and commercial activities.

As additional supporting evidence, we compared toluene concentrations measured by PTR-ToF-MS with those measured by GC-MS during NOAA mobile-laboratory observations in Los Angeles (Fig. R3). The two measurements were closely related (R^2 = 0.89, slope = 0.91, intercept = –0.06 ppbv), indicating that the PTR-ToF-MS signal at m/z 93 tracked toluene variability well across the sampled urban environment. This comparison does not exclude all possible interference, but it supports the interpretation that the m/z 93 signal in this region was dominated by toluene rather than by aromatic monoterpene fragments.

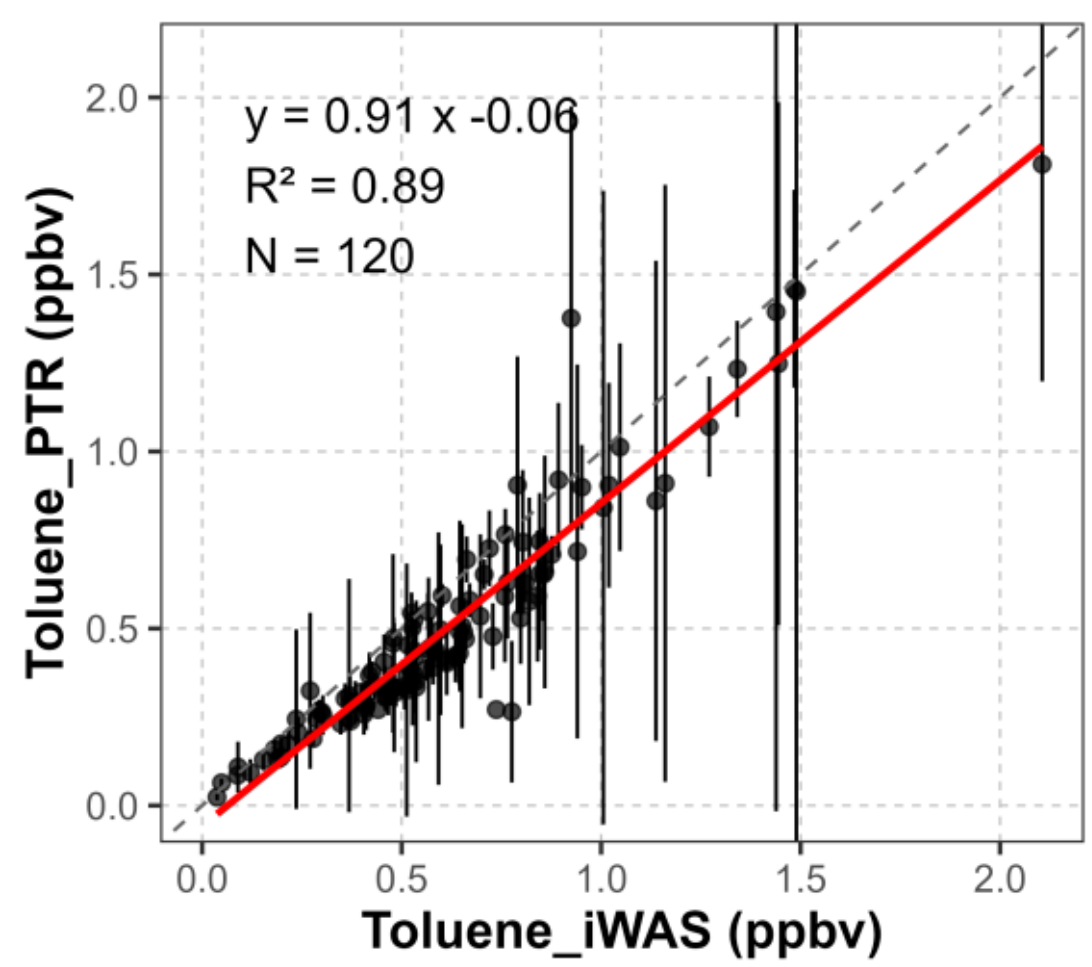

Fig. R3. Comparison of toluene concentrations measured by PTR-ToF-MS and GC-MS during NOAA mobile-laboratory observations in Los Angeles. Each point represents measurements averaged over the GC-MS sampling interval. Error bars denote the standard deviation of PTR-ToF-MS measurements within the corresponding averaging period. The red line indicates the least-squares regression, and the dashed line represents the 1:1 relationship.

To clarify, we have included the following contents in the revised manuscript and SI.

Lines 134–148: "Although previous studies indicate that proton-transfer-reaction time-of-flight mass spectrometry (PTR-ToF-MS) measurements of toluene may be subject to potential interferences[25], we do not expect such contributions to alter our main interpretation that the missing monoterpenoid emissions are primarily anthropogenic, because this interpretation is supported by consistent relationships across multiple tracers and independent emission indicators. We further analyzed proxies for different anthropogenic activities……We also examined correlations between monoterpenoid relative bias and fluxes of other chemical species (Fig. 2b). Significant negative correlations were found with aromatic and oxygenated VOCs such as xylene (r = –0.39, $p < 0.001$), acrolein (r = –0.32, $p < 0.001$), and nonanal (r = –0.31, $p < 0.001$), suggesting that the missing

monoterpenoid emissions are more strongly associated with solvent use (xylene)[17,27] and lipid oxidation or heat-related processes (nonanal and acrolein)[28]."

Minor comments

5. Lines 113-115. Elsewhere in the manuscript, octanal is mentioned as a tracer for cooking (line 160) and nonanal and acrolein are also associated with emissions from cooking (see manuscript ref 24). What does the correlation with relative bias look like for these tracers compared to the use of restaurant density?

Response: We examined correlations between monoterpenoid relative bias and fluxes of other chemical species (Fig. 2b), including several tracers associated with cooking emissions defined in previous studies. Significant negative correlations were found with oxygenated VOCs such as acrolein (r = –0.32, $p < 0.001$) and nonanal (r = –0.31, $p < 0.001$), as well as with aromatic compounds such as xylene (r = –0.39, $p < 0.001$). The octanal signal was available in the PTR-MS dataset, however, because it was not included in the calibration gas standards, the corresponding flux estimate is associated with greater uncertainty and was therefore not used as a primary quantitative tracer in this analysis.

To further investigate potential sources of nonanal, we examined the spatial distribution of nonanal concentrations from mobile-laboratory measurements (Fig. S5); acrolein concentrations were not available in the mobile measurements. Similar to octanal and isoprene, nonanal hotspots were observed near food industry facilities in industrial areas, suggesting that these emissions may be associated with food industry activities rather than restaurant cooking alone.

Taken together, these results indicate that the significant negative correlation between monoterpenoid relative bias and fluxes of nonanal and acrolein is more consistent with lipid oxidation or heat-related processes than with restaurant cooking, which was represented in the manuscript using restaurant density as a proxy.

To make this point clearer, we have elaborated the following descriptions in the revised manuscript to better distinguish restaurant cooking from other heat-related anthropogenic activities.

Lines 138–148: "……We further analyzed proxies for different anthropogenic activities. Decamethylcyclopentasiloxane (D5) flux and restaurant density were used as indicators of residential VCPs and restaurant cooking, respectively, two established anthropogenic sources of monoterpenoids (Figs. 2e–f)[26,27]. Neither showed a significant negative correlation with relative bias, suggesting that missing monoterpenoid emissions originate from anthropogenic activities beyond residential VCP and restaurant cooking sources. We also examined correlations between monoterpenoid relative bias and fluxes of other chemical species (Fig. 2b). Significant negative correlations were found with aromatic and oxygenated VOCs such as xylene (r = –0.39, $p < 0.001$), acrolein (r = –0.32, $p < 0.001$), and nonanal (r = –0.31, $p < 0.001$), suggesting that the missing monoterpenoid emissions are more strongly associated with solvent use (xylene)[17,27] and lipid oxidation or heat-related processes (nonanal and acrolein)[28]."

Lines 169–171: "Isoprene, octanal, and nonanal hotspots were also observed near these facilities (Fig. S5), suggesting additional anthropogenic contributions from food industry activities."

Lines 180–184: "In industrial areas, monoterpenoid concentrations were strongly correlated with isoprene (r = 0.56, p < 0.001), octanal (r = 0.45, p < 0.001), nonanal (r = 0.44, p < 0.001), benzaldehyde (r = 0.43, p < 0.001), and texanol (r = 0.33, p < 0.001), suggesting co-emission with compounds associated with food processing (octanal, nonanal, and isoprene) (Fig. S5) and industrial solvent (texanol)[26]."

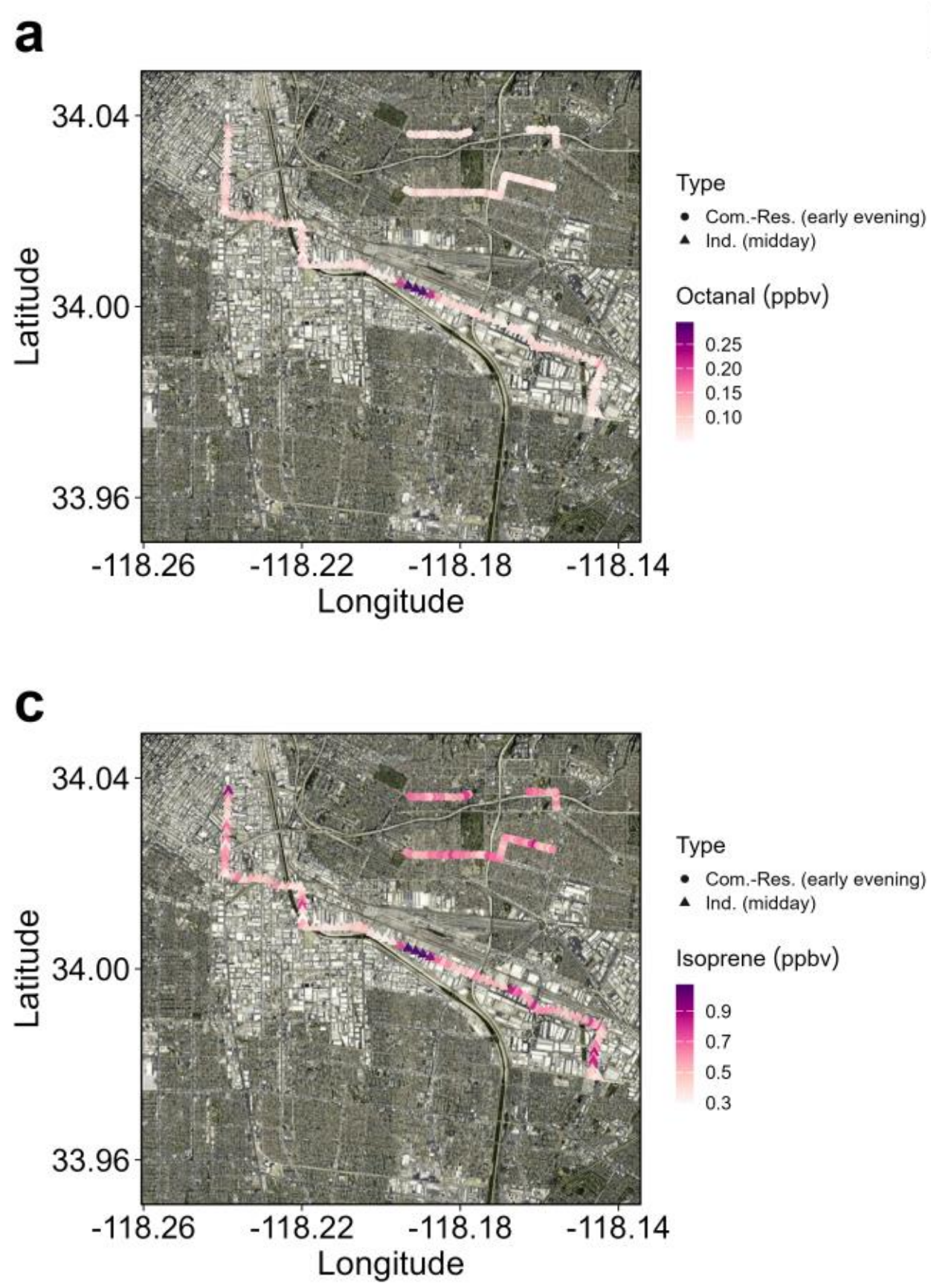

Fig. S5. Spatial distribution of octanal, nonanal, and isoprene concentrations from mobile-laboratory measurements. Marker shape denotes land-use type, with industrial areas characterized by white roofs.

6. Lines 80-97 I found this section difficult to follow and would recommend re-ordering to present the results of the old inventory approach first (Fig1a and 1b) and then move to describe the performance of the new approach (Fig1c and 1d). Presenting in that order seems more logical and would also mean figure panels are referred to in order of appearance.

Response: Revised as suggested. The section has been reordered to present the previous inventories first (Revised Figs. 1a–b), followed by the updated inventory (Revised Figs. 1c–d), with figure panels now discussed in order of appearance. See revised text below.

Lines 83–99: "Isoprene and total monoterpenoid emissions from inventories were evaluated against airborne flux measurements on a grid-to-grid (4×4 km^2) and time-resolved basis. For each cell, emissions were calculated as the sum of anthropogenic emissions and MEGAN-estimated biogenic emissions (Methods) (Figs. 1a–d). We first evaluated two previously developed inventory frameworks (Figs. 1a–b), including the combined MEGANv3.2$_{Default}$ + GRA^{2}PESv1.1 and CARB$_{Bio}$ + CARB$_{Anthro}$ inventories. Under the MEGANv3.2$_{Default}$ + GRA^{2}PESv1.1 framework, both isoprene and monoterpenoid emissions were largely underestimated, explaining only 3% and 7% of observed values (0.54 ± 0.37 mg m^{-2} h^{-1} for isoprene; 0.73 ± 0.39 mg m^{-2} h^{-1} for monoterpenoids), respectively. In comparison, the CARB emission framework showed an overestimation of isoprene by 90% and an underestimation of monoterpenoids by 76%.

We then evaluated the updated inventory developed in this study, which combines MEGANv3.2$_{Updated}$ with GRA^{2}PESv1.1 (Figs. 1c–d). Within this framework, biogenic sources accounted for nearly all isoprene emissions. Consistent with this, airborne flux measurements showed that high isoprene emissions were primarily associated with vegetated and remote areas (Fig. 1e), supporting the dominance of biogenic sources at the regional scale. The updated inventory reasonably captured the spatial distribution of isoprene ($R^2 = 0.37$) and explained 74% (0.40 ± 0.33 mg m^{-2} h^{-1}) of observed values, showing a substantial improvement compared with previous inventories (Fig. 1a)……"

7. Lines 80–97 – I am missing a clear, direct comparison between the standard MEGAN configuration (v3.2?) and the updated MEGAN version (v3.2_updated?). It is not clear whether the "standard" MEGAN used in this work corresponds to v3.2, or to the v3.0 referred to later on line 299. Am I correct in assuming that CARB_Bio essentially represents the standard MEGAN biogenic inventory? If so, then some of the most informative analysis would come from an explicit comparison of standard MEGAN (CARB_Bio) and MEGAN_updated against observations. Alternatively, if this is already what is shown by comparing panels 1a and 1c, given that CARB_anthro appears to include zero isoprene emissions, it would help if this were stated more explicitly. As noted above, the enhanced biogenic inventory is a real strength of this study, so clearly demonstrating how the updated MEGAN performs relative to the original is essential. Additionally, I would recommend restructuring the Materials and Methods so that the original MEGAN approach is introduced first, followed by the updated scheme, with a clear explanation of what has changed between the two.

Response: We agree that a direct comparison between standard and updated MEGAN configurations is important. In the original manuscript, we focused on comparisons between the CARB biogenic inventory (CARB$_{Bio}$) and our MEGANv3.2 simulations with updated vegetation inputs (MEGANv3.2$_{Updated}$). CARB$_{Bio}$ was generated using MEGANv3.0 with partially customized inputs (e.g., updated LAI), and therefore does not represent a fully standard MEGAN configuration. We also clarified that MEGANv3.2 and MEGANv3.0 followed the same modeling framework, with differences primarily related to input datasets.

To address this concern, we have now added comparisons with a standard MEGAN configuration. Specifically, we performed additional simulations using MEGANv3.2 with default vegetation inputs (MEGANv3.2$_{Default}$) and compared these with MEGANv3.2$_{Updated}$. This allows for a more direct assessment of how updated vegetation inputs influence BVOC emission estimates relative to a standard MEGAN setup.

In addition, we have now stated in the revised Fig. 1 caption that, because isoprene emissions from the CARB$_{Anthro}$ and GRA^{2}PESv1.1 anthropogenic inventories are minor, the comparison in CARB$_{Bio}$ + CARB$_{Anthro}$ and MEGANv3.2$_{Updated}$ + GRA^{2}PESv1.1 inventories largely reflects the performance of the biogenic isoprene emissions relative to observations.

We have incorporated these contents into the revised manuscript and clarified the relationships among CARB$_{Bio}$, MEGANv3.2$_{Default}$, and MEGANv3.2$_{Updated}$ to avoid ambiguity, see below.

Lines 86–99: "……We first evaluated two previously developed inventory frameworks (Figs. 1a–b), including the combined MEGANv3.2$_{Default}$ + GRA^{2}PESv1.1 and CARB$_{Bio}$ + CARB$_{Anthro}$ inventories. Under the MEGANv3.2$_{Default}$ + GRA^{2}PESv1.1 framework, both isoprene and monoterpenoid emissions were largely underestimated, explaining only 3% and 7% of observed values (0.54 ± 0.37 mg m^{-2} h^{-1} for isoprene; 0.73 ± 0.39 mg m^{-2} h^{-1} for monoterpenoids), respectively. In comparison, the CARB emission framework showed an overestimation of isoprene by 90% and an underestimation of monoterpenoids by 76%.

We then evaluated the updated inventory developed in this study, which combines MEGANv3.2$_{Updated}$ with GRA^{2}PESv1.1 (Figs. 1c–d)……The updated inventory reasonably captured the spatial distribution of isoprene (R^2 = 0.37) and explained 74% (0.40 ± 0.33 mg m^{-2} h^{-1}) of observed values, showing a substantial improvement compared with previous inventories (Fig. 1a)……"

Lines 295–304: "**CARB biogenic inventory.** The CARB 2021 biogenic inventory was generated using MEGANv3.0. For the CARB inventory, the default MEGAN3.0 datasets for plant growth form, ecotype, and emission factors are used. Leaf area index (LAI) for non-urban grid cells is based on the year-specific 8-day 500-m resolution MODIS Terra/Aqua combined product (MCD15A2H). Because the MODIS LAI product does not provide information for urban regions, urban LAI is estimated from the US Forest Service's Forest Inventory and Analysis (FIA) urban tree plot data, processed using i-Tree v6. Peak summertime urban LAI for different urban regions is estimated, and this peak value is then adjusted for each 8-day MODIS period based on the relative change in non-urban MODIS LAI across the state. Hourly meteorology inputs used to drive MEGANv3.0 simulations are provided by WRF model simulations, and stress factor adjustments are not considered."

Lines 305–330: "**Development of high-resolution biogenic emission inventory using MEGANv3.2.** In this study, we use MEGANv3.2 for biogenic emission simulations. MEGANv3.2

follows the same modeling framework as earlier versions (e.g., MEGANv3.0). The general framework of MEGAN (applicable to v3.0 and later versions) can be expressed as……

We performed MEGANv3.2 simulations with default vegetation inputs (i.e., the standard MEGANv3.2 configuration, $MEGANv3.2_{Default}$) and updated inputs (i.e., $MEGANv3.2_{Updated}$) to quantify the effects of vegetation input updates (Fig. 1). The comparison between these two simulations indicates that the improvement in BVOC emission estimates arises mainly from refined biogenic inputs of urban trees and landcover maps."

"**Fig. 1. Evaluation of terpenoid emissions against airborne flux observations. a, b, c, d,** Scatterplots comparing inventory estimates with aircraft flux observations for **(a, c)** isoprene and **(b, d)** monoterpenoids. For **a** and **b**, light purple points and regression lines indicate the combined $MEGANv3.2_{Default}$ + $GRA^2PESv1.1$ inventories, while blue points and regression lines indicate the combined $CARB_{Bio}$ + $CARB_{Anthro}$ inventories, reproduced following Pfannerstill et al. (2024)[14]. For **c** and **d**, purple points and regression lines indicate the combined $MEGANv3.2_{Updated}$ + $GRA^2PESv1.1$ inventories, while green points and regression lines represent the $MEGANv3.2_{Updated}$ biogenic inventory. In **c**, the two regressions largely overlap, reflecting the minor contribution of anthropogenic emissions to isoprene and the dominance of biogenic sources. **(e,g)** Spatial distribution of aircraft-derived isoprene and monoterpenoid flux. The yellow square highlights the Vernon and Commerce area. **(f, h)** Relative bias between inventory and airborne observations, calculated as (Inventory − Observation)/Observation. The yellow squares highlight the Colton and Long Beach area."

8. Line 87 and throughout – Rather than mg/m²/h, I recommend using the SI standard: mg m^{-2} h^{-1}.

Response: We thank the reviewer for this suggestion. All emission flux units have been updated to SI format (e.g., mg m^{-2} h^{-1}) throughout the manuscript.

9. Line 274 – change to "…other stresses on BVOC emissions in this study" – its not clear what the other factors being introduced are. I suggest you end that sentence at "study".

Response: Revised as suggested. The sentence now has been changed to "We did not consider the impact of water and other stresses on BVOCs."

10. Line 321 I would include the E/N that the instrument was operated with as this will influence fragmentation (see point above).

Response: The E/N value (130 Td) has now been included in the revised manuscript as suggested. As noted in our response to Comment 3, the PTR-ToF-MS was operated under stable reactor conditions (60 °C, 2.0 mbar), such that E/N remained effectively constant during the flights and fragmentation behavior was consistent.

11. Figs. 1, 2 and 4 Please change units from mg/m²/h to mg m^{-2} h^{-1}.

Response: Thanks and we have corrected it.

12. Figs S1, S2, S4 Please change units from mg/m^{2}*/h to mg m*$^{-2}$ *h*$^{-1}$.

Response: We have corrected it.

Response to Reviewer #2

This study by Zhang et al. examines the persistent gap between observed and modeled estimates of urban terpenoid emissions, focusing on the Greater Los Angeles area as a case study. Despite regulatory progress, urban regions like Los Angeles continue to face elevated levels of ozone and fine particulate matter, partly due to the incomplete characterization of volatile organic compound (VOC) sources. The research reveals that current emission inventories, such as those from the California Air Resources Board (CARB), significantly underestimate monoterpenoid emissions—by roughly 73%—when compared to direct airborne flux measurements. While the updated biogenic emission models, like MEGANv3.2, effectively capture the spatial distribution and magnitude of isoprene emissions, they fail to account for the observed flux levels of monoterpenoids, suggesting the presence of unaccounted or misclassified sources.

The study identifies a critical oversight in existing frameworks: previously unrecognized anthropogenic sources that mimic the temperature-dependent behavior typically associated with biogenic emissions. The work nicely explores the anthropogenic flux of terpenoids (in Great Los Angeles) from overlooked sources (ie. commercial activities and food industry) on a spatial basis (4 km - grid cell) by combining complementary approaches in a smart way: updated and highly-resolved emission inventory, flux measurements on board aircraft and mobile measurements. This work supports recent studies stressing that anthropogenic emissions of terpenoids which are temperature dependent as for their biogenic emissions, potentially leading to ambiguity in their origin attribution. Combining different approaches, proceeding step-by-step and detailed spatial analysis are very convincing to point out that the commercial activities and food industry are a significant source of terpenoids in Los Angeles, their temperature dependence. The specific use of proxies of various activities and of various nature (toluene, D5 siloxane, % of Tree Cover Fraction, temperature, restaurant density, bias calculation) strengthens the conclusions of the paper.

Response: We thank the reviewer for the positive and insightful comments. We appreciate the recognition of the discrepancy between observed and inventory-estimated emissions, as well as our integrated approach and identification of anthropogenic sources with biogenic-like temperature dependence. The manuscript has been revised to further clarify the methodology and discussion, and all responses and revisions are highlighted in blue and red, respectively.

1. A key insight from the study is the temperature dependence of these anthropogenic emissions, which closely resembles that of biogenic sources but is distinguished by a clear weekday–weekend variability—a temporal signature absent in purely biogenic emissions. This dual behavior not only complicates traditional methods of attributing emissions but also emphasizes the need to revise current inventories to explicitly include temperature-dependent anthropogenic sources. The study categorizes grid cells into "Inventory-accounted," "Mixed-source," and "Inventory-unexplained" types to better isolate and analyze these contributions. Notably, the "Inventory-unexplained" grid cells, which are dominated by these newly identified anthropogenic sources, exhibit monoterpenoid emission intensities that are four times higher than those in areas dominated by known sources.

Those emission sectors are not accounted for in the present emission inventory from CARB. The question of anthropogenic emissions of terpenoids have emerged since Mc Donalds work in 2018 on Volatile Chemical Products. It is now well established that terpenoids in urban environments are anthropogenic as shown by Borbon et al. (JGR, 2023) through a comprehensive overview of urban areas worldwide and more recently by Asfara et al in Los Angeles (GRL, 2026). In the present paper the authors only present terpenoid emissions through a temperature-dependence perspective but it provides a partial picture: I would recommend to include the above mentioned references. Please also note that there is the work by Farhat et al. (2024) in Dunkirk harbor, northern France who showed the industrial origin of terpenoids including food industry without going into such spatial details. In her following work Farhat also showed that anthropogenic terpenoids compete with biogenic terpenoids for SOA formation (Farhat et al., EST, 2025). This reference is cited in Line 121 but should be included in the introduction. Including those references would broaden the context and broaden its perspective.

Response: We thank the reviewer for this insightful comment and for highlighting relevant studies. We have revised the Introduction to better reflect the current understanding by incorporating anthropogenic terpenoid sources, including findings from Borbon et al. (2023), Asfara et al. (2026), and Farhat et al. (2024), which demonstrate the widespread presence of anthropogenic terpenoids in urban environments.

We also clarified that the focus of this study is not solely on the temperature dependence of terpenoid emissions, but on the combined effect of anthropogenic origin and biogenic-like temperature dependence. This dual behavior complicates source attribution and contributes to biases in current emission inventories, highlighting the need to represent temperature-dependent anthropogenic sources in inventory frameworks.

These revisions have been incorporated in the Introduction, see details below.

Lines 39–65: "A central challenge for cities like LA, where air pollution persists despite regulatory progress, is to identify the dominant sources of VOC emissions. During early stages of rapid urban development, industry and transportation sectors were the primary contributors to VOC emissions. As regulatory controls have reduced emissions from these sectors, the relative contribution of other sources has increased[7–12]. McDonald et al. (2018) highlights the increasing relative importance of volatile chemical products (VCPs), which now account for nearly half of fossil fuel-related VOC emissions in industrialized cities[9]. While these sources are primarily driven by population patterns, recent observations suggest that an additional class of urban VOC emissions is not fully captured by traditional source categories. In particular, temperature-sensitive VOC emissions, a feature usually considered biogenic, have been increasingly observed in urban environments[13,14]. For example, direct airborne flux measurements over LA demonstrate that terpenoid emissions, primarily isoprene and monoterpenoids (including monoterpenes and their oxygenated derivatives), show a strong temperature dependence and account for approximately 60% of total

emitted VOC OH reactivity and secondary organic aerosol (SOA) formation potential during summer[14].

Observational evidence across multiple cities further indicates that terpenoids are not exclusively biogenic but can also originate from anthropogenic activities such as traffic, VCPs, solvent use, and industrial processes[15–17]. This combination of anthropogenic origin and biogenic-like temperature dependence makes terpenoids challenging to attribute using conventional emission frameworks, in which anthropogenic emissions are typically treated as temperature-independent while biogenic emissions are parameterized as strongly temperature-dependent. As a result, these emissions are difficult to represent accurately in current emission inventories. For example, current major inventories, such as the California Air Resources Board (CARB) inventory, fail to capture the spatial variability of terpenoid emissions and substantially underestimate monoterpenoid emissions compared to airborne flux measurements[14]. Therefore, resolving this discrepancy requires not only identifying missing sources but also overcoming the limitations of current frameworks, which typically consider temperature-dependent emissions with biogenic origins. Addressing this mechanistic ambiguity is critical for effective air pollution control in LA and other similar regions."

2. However I have some suggestions and especially some clarification.

- Fig 1 looks incomplete: on Fig 1c the MEGAN+GRAPES purple points do not appear. Is it because they are hidden by the green color (MEGAN only) because of the biogenic origin of isoprene ? If so please add a comment in the legend.

Response: We have noted this point in the revised figure caption, where we stated that "In **c**, the two regressions largely overlap, reflecting the minor contribution of anthropogenic emissions to isoprene and the dominance of biogenic sources."

3. - The Reconciliation section is a little bit confused at least at the beginning: I suggest to add one or two sentences to explain the purpose of this section before introducing the categorizations.

Response: We thank the reviewer for this helpful suggestion. To clarify the purpose of the Reconciliation section, we have added an introductory statement explaining that isoprene and monoterpenoid emissions are influenced by different source mixtures, and therefore require different classification criteria. The added text is shown below.

Lines 198–204: "Isoprene and monoterpenoid emissions were influenced by different sources, so we applied source-specific indicators to categorize grid cells and reconcile observed and modeled fluxes. Our above analyses indicated that isoprene emissions were dominated by biogenic sources, with additional contributions from combustion-related anthropogenic activities, whereas monoterpenoid emissions arose from a broader mix of biogenic and multiple anthropogenic sources, including food industry and commercial activities. Therefore, different tracer-based criteria were used to classify grid cells for isoprene and monoterpenoids, respectively."

4. - The potential biases from other emission sources are not discussed: role of asphalte emissions from road pavement which are also temperature dependent (Khare et al., 2021; Salameh et al., 2024) is not discussed. The effect of traffic emissions around the mobile vehicle may also « contaminate » the measurements. A discussion on those potential biases would be welcome.

Response: We thank the reviewer for this helpful suggestion. We agree that other emission sources, including asphalt-related emissions from road pavement and possible contamination from nearby traffic during field measurements, should be considered when interpreting the monoterpenoid relative bias.

Based on the comprehensive evidence chain presented in this study, the discrepancies between inventories and observations for monoterpenoids are more consistently explained by emissions associated with food industry and small commercial activities. This interpretation is supported by (i) spatial patterns of fluxes and relative bias derived from airborne observations, (ii) consistent correlations with multiple tracers such as toluene, xylene, acrolein, and nonanal, and (iii) spatial co-location of monoterpenoid hotspots identified by mobile-laboratory measurements with specific facilities (e.g., food processing, laundries, florists, and car wash businesses). The first two lines of evidence provide regional scale constraints, and the third represents local scale source identification. Together, these observations indicate that commercial and industrial sources dominate the missing monoterpenoid emissions at the regional scale, and we do not find clear evidence for asphalt-dominated emissions. However, we agree that asphalt emissions are a temperature-dependent anthropogenic source that is not explicitly represented in current anthropogenic emission inventories. We have therefore revised the manuscript to highlight the importance of incorporating temperature dependence for certain anthropogenic sources such as asphalt-related emissions in future inventory development.

We also acknowledge that mobile-laboratory measurements may be influenced by nearby traffic emissions. However, our interpretation of the monoterpenoid relative bias is supported by consistent relationships across multiple tracers and independent emission indicators. In particular, no significant negative correlation was found between benzene flux, a tracer for traffic-related combustion emissions, and monoterpenoid relative bias (Revised Fig. 2b), suggesting that the missing monoterpenoid emissions are not primarily associated with traffic at the regional scale. In addition, source interpretation from the mobile-laboratory observations was not based on monoterpenoid hotspots alone, but on their co-occurrence with multiple tracers and their repeated spatial association with specific commercial and industrial facilities. This reduces, but does not fully eliminate, the possibility that individual hotspots were affected by transient traffic plumes. Nevertheless, because these mobile-laboratory observations were obtained in a limited area, source attribution based on the identified hotspots may not be fully representative of anthropogenic monoterpenoid sources across the broader Los Angeles region.

For clarity, we have added the following contents in the revised manuscript. Revised Fig. 2 has replaced the original Fig. 2 in the manuscript.

Lines 143–148: "……We also examined correlations between monoterpenoid relative bias and fluxes of other chemical species (Fig. 2b). Significant negative correlations were found with aromatic and oxygenated VOCs such as xylene (r = –0.39, p < 0.001), acrolein (r = –0.32, p < 0.001), and nonanal (r = –0.31, p < 0.001), suggesting that the missing monoterpenoid emissions are more strongly associated with solvent use (xylene)[17,27] and lipid oxidation or heat-related processes (nonanal and acrolein)[28]."

Lines 190–196: "We acknowledge that the above results are derived from high-resolution mobile-laboratory measurements obtained in a limited area (Vernon and Commerce). Similar VOC measurements in other parts of Los Angeles would be needed to draw more robust conclusions regarding contribution of different anthropogenic sources to monoterpenoids across the entire region. Nonetheless, the combination of airborne flux measurements, which provide regional scale constraints, and high-resolution mobile-laboratory observations, which enable local source identification, offers valuable complementary insights into terpenoid emission sources."

Lines 256–261: "……While recent studies have highlighted the climate penalty of temperature-driven anthropogenic emissions[13], our findings point to a broader limitation in current emission frameworks in that temperature-dependent processes in certain anthropogenic sectors may not be adequately captured. This limitation may also extend to other plausible sources, such as asphalt-related emissions from road pavement, which have been shown to increase with temperature and could contribute to urban air pollution[31,32]……"

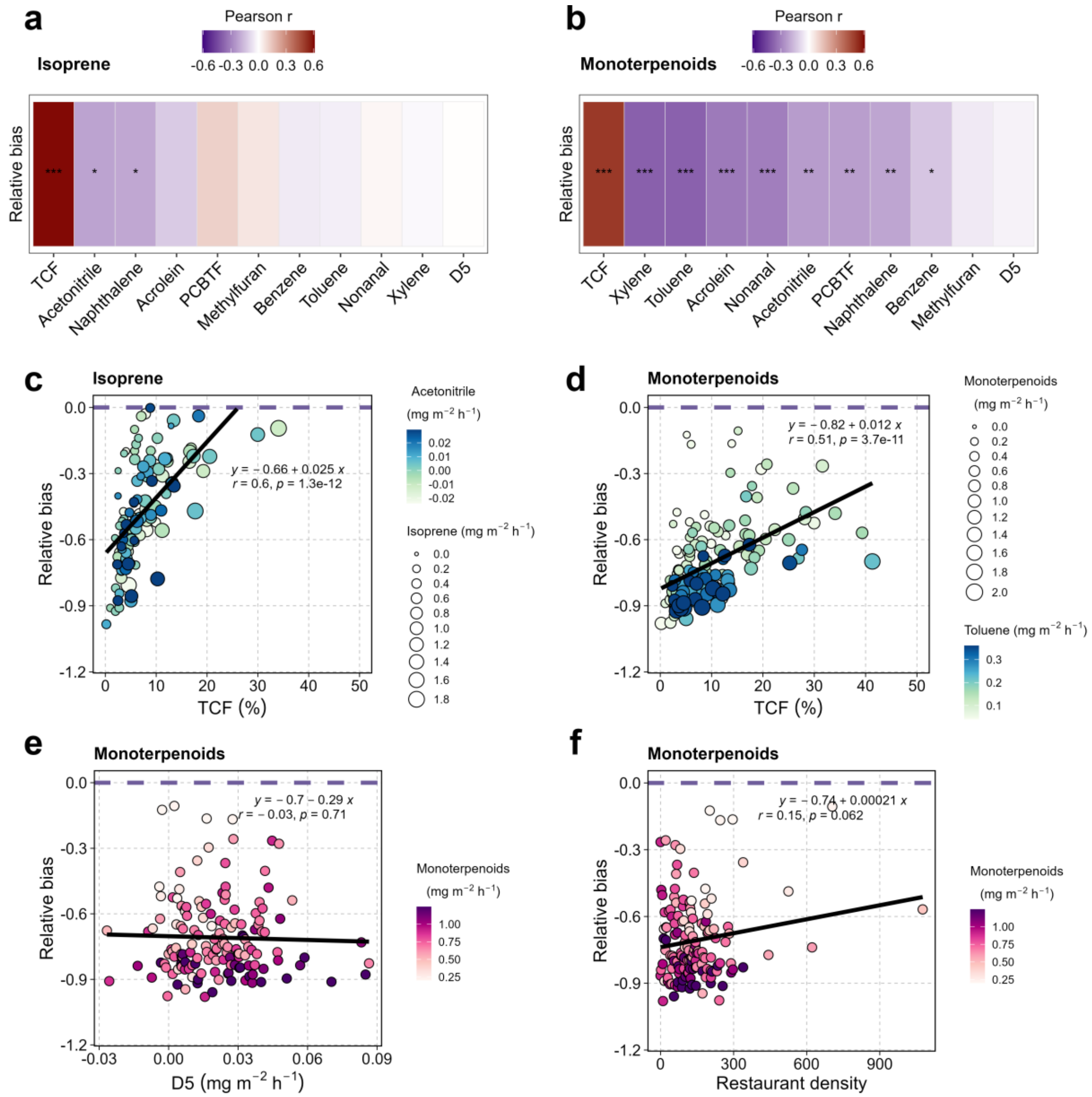

Revised Fig. 2. Relationship between relative bias of the combined MEGANv3.2$_{\text{Updated}}$ + GRA^{2}PESv1.1 terpenoid inventories and various emission indicators. a,b, Pearson correlations between relative bias in **(a)** isoprene and **(b)** monoterpenoid fluxes and tree cover fraction (TCF, %) and airborne fluxes of selected chemical species. Colors indicate pearson correlation coefficients (r), and asterisks denote statistical significance (*$p < 0.1$; **$p < 0.01$; ***$p < 0.001$). **c,d,** Isoprene relative bias and monoterpenoid relative bias versus TCF, colored by (**c**) acetonitrile flux and (**d**) toluene flux. Marker size denotes aircraft-derived isoprene and monoterpenoid flux, and marker color indicates the co-located emission proxy in each panel. **e,f,** Monoterpenoid relative bias versus anthropogenic indicators, including (**e**) D5 flux and (**f**) restaurant density. Restaurant locations in Los Angeles were obtained via the Google Maps API, and grid-cell counts were used as a proxy for cooking intensity. Solid black lines represent least-squares fits, with r and p-values indicated.

5. - In the Material and Method it would be valuable to explain how the emissions of terpenoids are established in the emission inventory in addition to the used datasets.

Response: We thank the reviewer for this helpful suggestion. In the revised manuscript, we have clarified how terpenoid emissions are established in each biogenic emission inventory ($CARB_{Bio}$, $MEGANv3.2_{Default}$, and $MEGANv3.2_{Updated}$) by providing a more explicit description of the input datasets used. These clarifications make it more explicit how differences in input datasets across inventories lead to differences in terpenoid emission estimates.

Lines 295–304: "**CARB biogenic inventory.** The CARB 2021 biogenic inventory was generated using MEGANv3.0. For the CARB inventory, the default MEGAN3.0 datasets for plant growth form, ecotype, and emission factors are used. Leaf area index (LAI) for non-urban grid cells is based on the year-specific 8-day 500-m resolution MODIS Terra/Aqua combined product (MCD15A2H). Because the MODIS LAI product does not provide information for urban regions, urban LAI is estimated from the US Forest Service's Forest Inventory and Analysis (FIA) urban tree plot data, processed using i-Tree v6. Peak summertime urban LAI for different urban regions is estimated, and this peak value is then adjusted for each 8-day MODIS period based on the relative change in non-urban MODIS LAI across the state. Hourly meteorology inputs used to drive MEGANv3.0 simulations are provided by WRF model simulations, and stress factor adjustments are not considered."

Lines 305–330: "**Development of high-resolution biogenic emission inventory using MEGANv3.2.** In this study, we use MEGANv3.2 for biogenic emission simulations. MEGANv3.2 follows the same modeling framework as earlier versions (e.g., MEGANv3.0). The general framework of MEGAN (applicable to v3.0 and later versions) can be expressed as……

We performed MEGANv3.2 simulations with default vegetation inputs (i.e., the standard MEGANv3.2 configuration, $MEGANv3.2_{Default}$) and updated inputs (i.e., $MEGANv3.2_{Updated}$) to quantify the effects of vegetation input updates (Fig. 1). The comparison between these two simulations indicates that the improvement in BVOC emission estimates arises mainly from refined biogenic inputs of urban trees and landcover maps."

Lines 331–343: "**Updated vegetation inputs for MEGANv3.2 simulations.** LAI inputs were derived from the Copernicus Global Land Service (CGLS) Collection 300-m datasets (https://land.copernicus.eu/en/products/vegetation/leaf-area-index-300m-v1.0). For urban landscapes in the study region, ecotypes and tree species composition were derived from a zip-code–level urban tree inventory for California[40] (https://lookerstudio.google.com/reporting/880d448d-de26-48d3-b563-0c6317e456e4/page/jWHKB) and used to construct the MEGAN ecotype map layer and associated VCLUT. Growth form fraction inputs were derived from WorldCover 10-m data, where grid cells dominated by a specific vegetation type were assigned a fraction of 100%, while all others were set to 0%. To validate the accuracy of WorldCover data, we compared city-average vegetation cover fraction and tree cover fraction in major California cities against estimates from i-Tree

(https://www.itreetools.org/) and Google Environmental Insights Explorer (GEIE; https://insights.sustainability.google/) virtual observations (Fig. S8). The strong agreement with field measurements supports the use of WorldCover 10-m data as a GF input for MEGANv3.2."

6. The implications of these findings extend beyond Los Angeles. Given that food industry and commercial activities are intrinsic to urbanization worldwide, the study suggests that these underrepresented sources likely contribute to air quality challenges in other megacities. Addressing these sources could provide a tangible, previously unrecognized opportunity to mitigate urban ozone and secondary organic aerosol formation. Finally, this research calls for improvements in both anthropogenic and biogenic emission modeling. For anthropogenic sources, the study advocates for the inclusion of temperature-dependent categories to better capture their behavior under a warming climate. For biogenic sources, it highlights the need to refine models to account for urban vegetation stress, non-foliar emissions, and updated emission factors, which are currently underestimated by approximately 30%. By integrating these insights, air quality management frameworks can more accurately attribute emissions and develop targeted strategies to reduce pollution in urban environments

To conclude I recommand this paper for publication once the above mentionned items have been taken into account.

Response: We thank the reviewer for this encouraging assessment and for recognizing the broader implications of our findings beyond Los Angeles. We appreciate the reviewer's positive evaluation of the study and the recommendation for publication once the above points are addressed.

In the revised manuscript, we have further clarified the broader relevance of these findings and strengthened the discussion of their implications for both anthropogenic and biogenic emission modeling. In particular, we have now elaborated the respective roles of temperature sensitivity and activity-related temporal variability for anthropogenic sources. We have also revised the discussion of biogenic discrepancy to better reflect the evidence presented in the manuscript. The revisions are detailed below.

Lines 255–266: "Understanding the sources, activity profile, and temperature sensitivity of terpenoid emissions offers guidance for urban air quality management. While recent studies have highlighted the climate penalty of temperature-driven anthropogenic emissions[13], our findings point to a broader limitation in current emission frameworks in that temperature-dependent processes in certain anthropogenic sectors may not be adequately captured. This limitation may also extend to other plausible sources, such as asphalt-related emissions from road pavement, which have been shown to increase with temperature and could contribute to urban air pollution[31,32]. Re-attributing these hidden anthropogenic emissions and incorporating both activity-related temporal variability and temperature dependence into emission inventories may improve the representation of anthropogenic VOC emissions. In particular, distinguishing between temperature-independent, temperature-dependent, and strongly temperature-dependent

anthropogenic emissions could help improve the representation of urban VOC emissions in chemical transport models under a changing climate."

Lines 273–279: "Our results also provide insights into refining the biogenic emission model. The substantial improvement in isoprene estimates after updating urban vegetation inputs demonstrates the importance of accurately representing urban land cover and vegetation composition. However, residual inventory–measurement differences remain, especially for monoterpenoids. Although these differences cannot be attributed solely to biogenic emissions, they highlight remaining uncertainties in urban BVOC modeling, including canopy microenvironment, emission factors, and missing processes such as vegetation stress responses and non-foliar emissions……"

References

1. Pfannerstill, E. *et al.* Temperature-dependent emissions dominate aerosol and ozone formation in Los Angeles. *Science* **384**, 1324–1329 (2024).
2. Diao, L. *et al.* Discrepancies between modeled and observed nocturnal isoprene in an urban environment and the possible causes: A case study in Houston. *Atmos. Res.* **181**, 257–264 (2016).
3. Al-Naiema, I. M. *et al.* Source apportionment of fine particulate matter in Houston, Texas: insights to secondary organic aerosols. *Atmos. Chem. Phys.* **18**, 15601–15622 (2018).
4. Wu, W. *et al.* Temperature-Dependent evaporative anthropogenic VOC emissions significantly exacerbate regional ozone pollution. *Environ. Sci. Technol.* **58**, 5430–5441 (2024).
5. Qin, M. *et al.* Increased urban ozone in heatwaves due to temperature-induced emissions of anthropogenic volatile organic compounds. *Nat. Geosci.* **18**, 50–56 (2025).
6. Coggon, M. M. *et al.* Identifying and correcting interferences to PTR-ToF-MS measurements of isoprene and other urban volatile organic compounds. *Atmos. Meas. Tech.* **17**, 801–825 (2024).
7. Tani, A. Fragmentation and reaction rate constants of terpenoids determined by proton transfer reaction-mass spectrometry. *Environ. Control Biol.* **51**, 23–29 (2013).

Response to Reviewers' Comments

The reviewer's comments are listed below in *black italics*, and our responses are highlighted in blue.

Response to Reviewer #1:

I have reviewed the revised manuscript and can see that the authors have carefully considered my comments and undertaken substantial additional analysis and revisions. I commend them for their thorough and thoughtful approach. I am satisfied that all of my concerns have been addressed and have no further comments. I am therefore happy to recommend this manuscript for publication.

Response: We thank the reviewer for the positive assessment and recommendation. We are pleased that the reviewer found that their concerns have been addressed. No further changes were required in response to this comment.

Review of Zhang et al. : *Reconciling observed and modeled estimates of urban terpenoid emissions submitted to Nature Communications*

This study by Zhang et al. examines the **persistent gap between observed and modeled estimates of urban terpenoid emissions, focusing on the Greater Los Angeles area as a case study**. Despite regulatory progress, urban regions like Los Angeles continue to face elevated levels of ozone and fine particulate matter, partly due to the incomplete characterization of volatile organic compound (VOC) sources. The research reveals that current emission inventories, such as those from the California Air Resources Board (CARB), significantly underestimate monoterpenoid emissions—by roughly 73%—when compared to direct airborne flux measurements. While the updated biogenic emission models, like MEGANv3.2, effectively capture the spatial distribution and magnitude of isoprene emissions, they fail to account for the observed flux levels of monoterpenoids, suggesting the presence of unaccounted or misclassified sources.

The study identifies a critical oversight in existing frameworks: **previously unrecognized anthropogenic sources** that mimic the temperature-dependent behavior typically associated with biogenic emissions. The work nicely explores the anthropogenic flux of terpenoids (in Great Los Angeles) from overlooked sources (ie. commercial activities and food industry) on a **spatial basis** (4 km - grid cell) by combining complementary approaches in a smart way : updated and highly-resolved emission inventory, flux measurements on board aircraft and mobile measurements. This work supports recent studies stressing that anthropogenic emissions of terpenoids which are temperature dependent as for their biogenic emissions, potentially leading to ambiguity in their origin attribution. Combining different approaches, proceeding step-by-step and detailed spatial analysis are very convincing to point out that the commercial activities and food industry are a significant source of terpenoids in Los Angeles, their temperature dependence. The specific use of proxies of various activities and of various nature (toluene, D5 siloxane, % of Tree Cover Fraction, temperature, restaurant density, bias calculation) strengthens the conclusions of the paper

A key insight from the study is the temperature dependence of these anthropogenic emissions, which closely resembles that of biogenic sources but is distinguished by a clear weekday–weekend variability—a temporal signature absent in purely biogenic emissions. This dual behavior not only complicates traditional methods of attributing emissions but also emphasizes the need to revise current inventories to explicitly include temperature-dependent anthropogenic sources. The study categorizes grid cells into "Inventory-accounted," "Mixed-source," and "Inventory-unexplained" types to better isolate and analyze these contributions. Notably, the "Inventory-unexplained" grid cells, which are dominated by these newly identified anthropogenic sources, exhibit monoterpenoid emission intensities that are four times higher than those in areas dominated by known sources. Those emission sectors are not accounted for in the present emission inventory from CARB. The question of anthropogenic emissions of terpenoids have emerged since Mc Donalds work in 2018 on Volatile Chemical Products. It is now well established that terpenoids in urban environments are anthropogenic as shown by Borbon et al. (JGR, 2023) through a comprehensive overview of urban areas worldwide and more recently by Asfara et al in Los Angeles (GRL, 2026). In the present paper the authors only present terpenoid emissions through a temperature-dependence perspective but it provides a partial picture : I would

recommend to include the above mentioned references. Please also note that there is the work by Farhat et al. (2024) in Dunkirk harbor, northern France who showed the industrial origin of terpenoids including food industry without going into such spatial details. In her following work Farhat also showed that anthropogenic terpenoids compete with biogenic terpenoids for SOA formation (Farhat et aL., EST, 2025). This reference is cited in Line 121 but should be included in the introduction. Including those references would broaden the context and broden its perspective.

However I have some suggestions and especially some clarification

- Fig 1 looks incomplete : on Fig 1c the MEGAN+GRAPES purple points do not appear. Is it because they are hidden by the green color (MEGAN only) because of the biogenic origin of isoprene ? If so please add a comment in the legend
- The *Reconciliation* section is a little bit confused at least at the beginning : I suggest to add one or two sentences to explain the purpose of this section before introducing the categorizations
- The potential biases from other emission sources are not discussed : role of asphalte emissions from road pavement which are also temperature dependent (Khare et al., 2021 ; Salameh et al., 2024) is not discussed. The effect of traffic emissions around the mobile vehicle may also « contaminate » the measurements. A discussion on those potential biases would be welcome.
- In the *Material and Method* it would be valuable to explain how the emissions of terpenoids are established in the emission inventory in addition to the used datasets.

The implications of these findings extend beyond Los Angeles. Given that food industry and commercial activities are intrinsic to urbanization worldwide, the study suggests that these underrepresented sources likely contribute to air quality challenges in other megacities. Addressing these sources could provide a tangible, previously unrecognized opportunity to mitigate urban ozone and secondary organic aerosol formation.

Finally, this research calls for improvements in both anthropogenic and biogenic emission modeling. For anthropogenic sources, the study advocates for the inclusion of temperature-dependent categories to better capture their behavior under a warming climate. For biogenic sources, it highlights the need to refine models to account for urban vegetation stress, non-foliar emissions, and updated emission factors, which are currently underestimated by approximately 30%. By integrating these insights, air quality management frameworks can more accurately attribute emissions and develop targeted strategies to reduce pollution in urban environments

To conclude I recommand this paper for publication once the above mentionned items have been taken into account.
